# Clinical Risk Factors and Inflammatory Markers as Predictors of Postoperative Delirium in Patients Undergoing Acute Type A Aortic Dissection Surgery

**DOI:** 10.3390/jcm15208090

**Published:** 2026-10-18

**Authors:** Xiantong Cao, Botao Wang, Huan Yang, Yujing Wu, Yongjian Zhang, Liang Zhong, Tao Ma, Tao Shi, Zhanqin Zhang

**Affiliations:** 1 Department of Cardiovascular Surgery, First Affiliated Hospital of Xi’an Jiaotong University, No. 277 Yanta West Road, Xi’an 710061, China; caoxt759525@126.com (X.C.); 2548wbt@stu.xjtu.edu.cn (B.W.); yanghuan@xjtufh.edu.cn (H.Y.); yongjianchuang@163.com (Y.Z.); drlzhong@163.com (L.Z.); mataodr@163.com (T.M.); shi2009tao@163.com (T.S.); 2 Department of Emergency Medicine, First Affiliated Hospital of Xi’an Jiaotong University, No. 277 Yanta West Road, Xi’an 710061, China; lanyewyjcxt@163.com

**Keywords:** acute type A aortic dissection, postoperative delirium, predictive nomogram, systemic inflammation response index, procalcitonin, interleukin-6

## Abstract

**Background:** Postoperative delirium (POD) is a common and serious neurological complication following acute type A aortic dissection (ATAAD) repair, contributing to increased morbidity and mortality. Given the critical role of systemic inflammation in POD pathogenesis, this study aimed to evaluate perioperative inflammatory profiles in patients undergoing ATAAD repair and to develop a predictive nomogram for early POD risk stratification. **Methods:** This retrospective, single-center study included 355 patients who underwent total arch replacement for ATAAD. Serial inflammatory markers were assessed across multiple perioperative time points. A two-stage variable selection process, incorporating variance inflation factor (VIF) and the Boruta algorithm, was utilized to identify robust predictors. A predictive nomogram was constructed via multivariable logistic regression. Model performance was assessed using the area under the receiver operating characteristic curve (AUC), calibration plots, and decision curve analysis (DCA). Internal validation was performed using 1000 bootstrap resamples. **Results:** POD occurred in 29.86% of the cohort. Patients who developed POD had higher inflammatory marker levels at several individual postoperative time points compared to the non-delirium group. The final predictive model integrated 11 key variables: age, EuroSCORE I, CPB time, SIRI_6h, PLR_6h, IL-10_6h, IL-8_6h, IL-8_pre, IL-6_6h, IL-6_pre, and PCT_6h. The nomogram demonstrated acceptable internal discrimination with an AUC of 0.835 (95% CI: 0.794–0.876) and an optimism-corrected AUC of 0.811. Notably, IL-6_6h and PCT_6h emerged as strong independent risk factors, while SIRI_6h demonstrated an inverse association with POD. Calibration and DCA analyses confirmed the model’s acceptable performance and potential clinical utility, though independent external validation remains essential prior to any clinical translation. An exploratory, web-based risk calculator was developed to facilitate bedside implementation. **Conclusions:** This study develops and internally validates an exploratory biomarker-based prediction model for POD in ATAAD patients. By integrating early inflammatory profiles with established clinical variables, this preliminary model provides a preliminary foundation for early risk stratification, though rigorous prospective multicenter external validation is required before real-world clinical implementation can be considered.

## 1. Introduction

Acute type A aortic dissection (ATAAD) is a life-threatening cardiovascular emergency with substantial perioperative mortality and morbidity [1,2,3]. Despite ongoing advances in surgical techniques and extracorporeal circulation management, total arch replacement remains a highly complex procedure [1,2]. These interventions frequently necessitate hypothermic circulatory arrest to ensure cerebral protection and provide an adequate operative field [4,5]; however, the extent of repair and the use of deep hypothermia are inherently linked to prolonged cardiopulmonary bypass (CPB), cross-clamp, and circulatory arrest durations [4,6]. These factors collectively contribute to profound operative stress and a high incidence of postoperative complications. Postoperative delirium (POD) is among the most concerning neurological complications after ATAAD repair. In large ATAAD series, POD occurred in 31.8% of patients after modified triple-branched stent graft implantation and 46.8% in a recent biomarker study, underscoring its high frequency in this population [7,8]. Clinically, POD is characterized by fluctuating attention, disorganized thinking, and altered consciousness [7,8]. Its occurrence is independently associated with prolonged mechanical ventilation, extended ICU and hospital stays, increased healthcare costs, and higher in-hospital mortality, making it a major determinant of postoperative recovery and short-term prognosis in ATAAD patients [7].

The pathophysiology of POD after ATAAD repair is multifactorial and remains incompletely understood. However, accumulating evidence increasingly supports the inflammatory hypothesis, whereby surgical trauma and perioperative systemic inflammation contribute to POD through blood–brain barrier (BBB) disruption and subsequent neuroinflammation [9,10,11]. Mechanistically, tissue injury activates innate immune pathways and promotes the release of pro-inflammatory mediators, while BBB dysfunction permits peripheral cytokines and immune cells to access the central nervous system, thereby amplifying microglial activation and neuronal dysfunction [9,10,11]. In the context of ATAAD, patients may be uniquely susceptible to this process due to the hyperacute nature of the dissection, the frequent presence of cerebral or systemic malperfusion, and the requirement for urgent, highly invasive surgery—all of which exacerbate inflammatory stress and cerebral vulnerability.

Clinical risk factors for POD in this population are well-documented, with advanced age, renal insufficiency, prolonged CPB duration, and postoperative electrolyte disturbances being frequently cited [12,13,14]. Nevertheless, the clinical utility of current risk-prediction models remains limited. Systematic reviews of cardiovascular surgery cohorts indicate that existing models often lack robust external validation and fail to incorporate the heightened inflammatory burden characteristic of ATAAD [15,16]. While individual biomarkers—such as interleukin-6 (IL-6), interleukin-8 (IL-8), C-reactive protein (CRP), and various systemic inflammatory indices—have shown significant associations with POD, there is a paucity of clinical tools that systematically integrate these inflammatory profiles with established perioperative predictors [14,17,18]. To address these critical gaps, this study aims to develop and internally validate an exploratory clinical prediction model for POD in patients undergoing ATAAD repair. By integrating a broad panel of perioperative inflammatory markers (IL-6, IL-8, interleukin-10 (IL-10), procalcitonin (PCT), and systemic inflammatory indices) with standard clinical variables, we seek to construct a promising nomogram to assist early, personalized risk stratification. Such an approach may provide a foundation for future targeted preventive strategies, pending rigorous external validation.

## 2. Materials and Methods

### 2.1. Study Design and Participants

**Figure 1:**
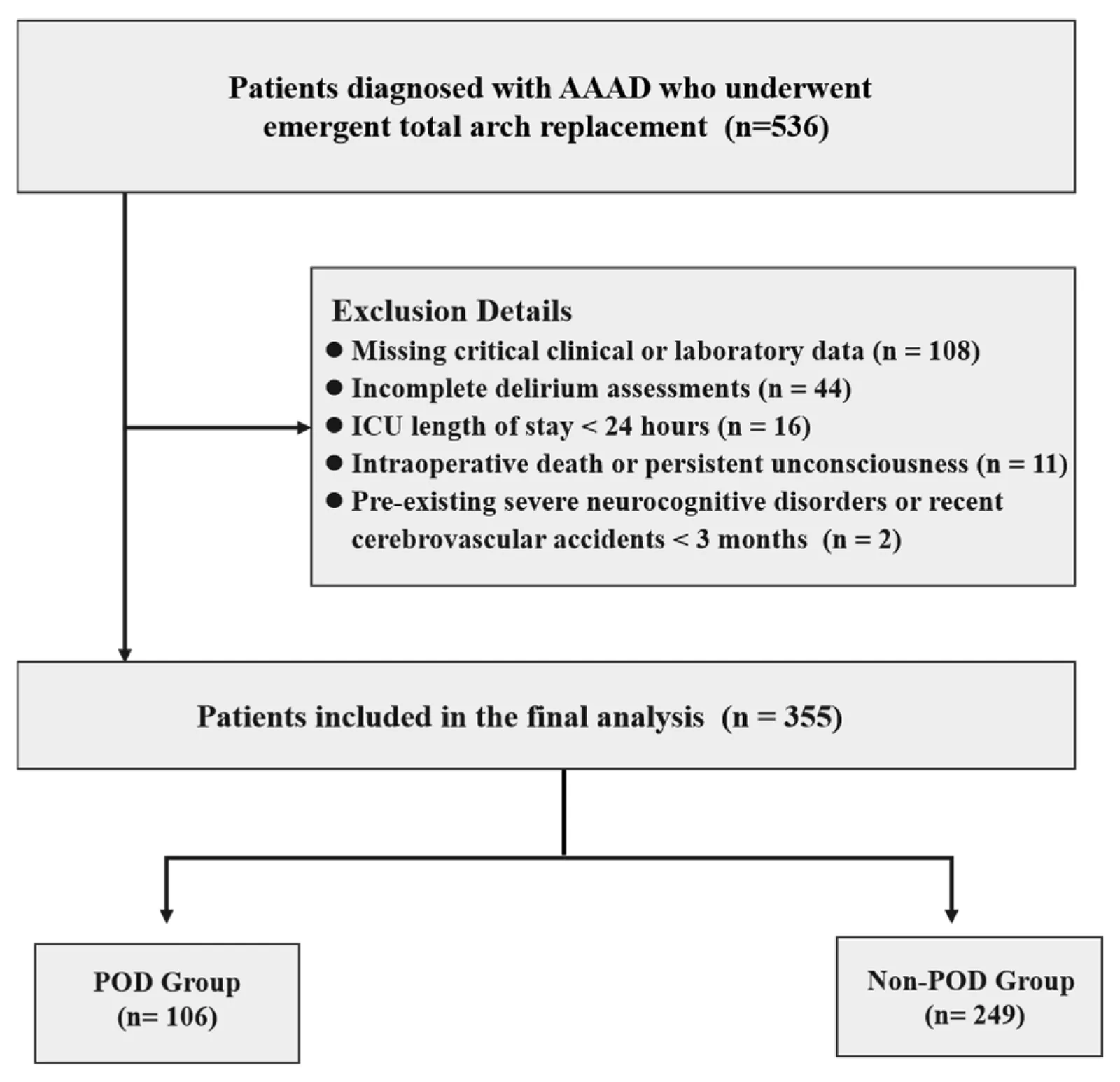
Flow chart of study population and groups.

This retrospective, single-center observational study was conducted at the First Affiliated Hospital of Xi’an Jiaotong University. We included consecutive patients diagnosed with ATAAD who underwent emergent total arch replacement between March 2020 and December 2022. The study flow is depicted in Figure 1. Of 536 initially screened patients, 355 met the inclusion criteria for the final complete-case analysis. Patients were excluded based on the following criteria: incomplete delirium assessments (*n* = 44), pre-existing severe neurocognitive disorders or acute/recent cerebrovascular accidents (<3 months) (*n* = 2), intraoperative death or persistent postoperative unconsciousness (*n* = 11), intensive care unit (ICU) length of stay less than 24 h (*n* = 16), and missing critical clinical or laboratory data (*n* = 108). A formal, a priori sample size calculation was not performed; rather, the sample size was determined by the maximum available cohort of consecutive eligible patients treated during the specified study period.

Missing values in the candidate predictors were handled using multiple imputation by fully conditional specification (FCS), implemented via IterativeImputer in scikit-learn; m = 20 imputed datasets were generated by running the FCS algorithm 20 times with different random seeds, with all 11 candidate predictors included and the outcome variable (which was not missing) included as an auxiliary variable to preserve the predictor–outcome relationship, as recommended for imputation in prediction-model research. Continuous and binary variables were imputed using Bayesian ridge regression as implemented in IterativeImputer, with sample_posterior=True to ensure between-imputation variability; 30 iterations were used per imputation, and convergence was assessed by inspection of trace plots of the imputed values across iterations, with no systematic trends observed after 15 iterations. Regression coefficients and their variances were combined across the 20 imputed datasets using Rubin’s rules, and for AUC, estimates were combined on the logit scale and back-transformed; in each Bootstrap sample, missing values were imputed afresh using the same FCS procedure, and the full model-fitting procedure was repeated, with performance estimates combined across Bootstrap samples and imputations using Rubin’s rules. To evaluate potential selection bias, baseline characteristics and clinical outcomes were compared between the included cohort and patients excluded due to missing data. Furthermore, a multiple-imputation sensitivity analysis was performed to assess whether the complete-case results were materially altered by the missing-data handling. All candidate predictors, including inflammatory markers measured at 6 h postoperatively, were systematically recorded.

### 2.2. Ethics Statement

The study protocol was approved by the Institutional Ethics Committee of the First Affiliated Hospital of Xi’an Jiaotong University (No: XJTU1AF2026LSYY-0270). Due to the retrospective nature of the study and the use of de-identified, anonymized data, the requirement for informed consent was waived by the committee.

### 2.3. Delirium Assessment

The primary outcome POD was assessed using the confusion assessment method for the ICU (CAM-ICU), a gold-standard diagnostic tool for critical care settings. Delirium surveillance was primarily conducted via routine assessments performed twice daily from postoperative hour 6 through postoperative day 7. In addition to routine monitoring, on-demand evaluations were performed whenever patients exhibited acute breakthrough neurological changes or fluctuations. The richmond agitation-sedation scale (RASS) was evaluated prior to each CAM-ICU assessment. Assessments were restricted to patients who were adequately responsive. Patients in a state of deep sedation or unresponsiveness (RASS score ≤ −4) were deemed untestable by CAM-ICU at that time point, and their assessments were deferred until lightening of sedation. A positive diagnosis of delirium required the presence of features 1 (acute onset/fluctuation) and 2 (inattention), accompanied by either feature 3 (disorganized thinking) or feature 4 (altered level of consciousness). All assessments were performed by trained ICU physicians to ensure consistency. Although this is a retrospective study, the 6-h postoperative blood sampling window chronologically preceded the clinical documentation of POD onset in all cases, providing a stable baseline for early risk prediction. Standardized perioperative sedation and analgesia protocols were implemented using propofol, dexmedetomidine, and opioids.

### 2.4. Data Collection and Inflammatory Markers

Demographic, operative, and laboratory data were extracted from the electronic medical record system. Preoperative risk was evaluated using the European system for cardiac operative risk evaluation (EuroSCORE) I, which was recorded as a standard clinical score upon admission. Blood samples were collected at fixed perioperative time points: preoperatively, and strictly at 6 h postoperatively, as well as on postoperative day 3 and postoperative day 5. serum was isolated immediately by centrifugation 3000 rpm for 10 min at 4 °C and stored at −80 °C in dedicated aliquots to prevent degradation, with no sample undergoing more than one freeze–thaw cycle prior to analysis. Systemic inflammatory indices were calculated from complete blood counts as follows: ① NLR: Neutrophils/Lymphocytes; ② PLR: Platelets/Lymphocytes; ③ SII: (Platelets × Neutrophils)/Lymphocytes; ④ SIRI: (Neutrophils × Monocytes)/Lymphocytes. Twelve serum cytokines (interleukin−1β (IL−1β), interleukin−2 (IL−2), interleukin−4 (IL−4), interleukin−5 (IL−5), IL−6, IL−8, IL−10, interleukin−12 (IL−12), interleukin−17 (IL−17), interferon-alpha (IFN−α), interferon-gamma (IFN−γ), tumor necrosis factor (TNF)) were quantified using the cytometric bead array (CBA) assay performed by the institutional clinical laboratory following standard operating procedures. Flow cytometry was performed for signal acquisition, and FCAP Array V3 software was used to calculate cytokine concentrations based on standard curves. All assays met the hospital’s strict clinical laboratory quality control standards, and laboratory personnel were strictly blinded to patients’ POD status and clinical outcomes. All continuous inflammation and immune biomarkers (specifically, SIRI, PLR, IL-10, and IL-8 at D1 and pre-op, IL-6 at D1 and pre-op, and PCT at D1) exhibited right-skewed distributions and were log-transformed using ln(x+1) prior to model fitting. Clinical baseline traits (age, EuroSCORE I, and CPB time) were modeled in their original clinical units.

### 2.5. Model Development

#### 2.5.1. Feature Selection and Collinearity Control

To build a robust and parsimonious predictive model while avoiding the limitations of traditional, univariate *p*-value based screening thresholds (which often discard variables with complex or non-linear predictive value), we adopted a multi-stage variable selection strategy. First, we calculated a pairwise correlation matrix specifically among perioperative inflammatory markers to better understand their interrelationships and network structure. Next, to quantitatively rule out severe multicollinearity, we computed the variance inflation factor (VIF) for all candidate variables and applied a strict retention threshold of VIF < 5. Finally, the Boruta algorithm, a robust feature selection wrapper built on Random Forest, was applied to all candidate variables to objectively identify features exhibiting significant predictive importance for POD.

#### 2.5.2. Multivariable Modeling and Justification of Variable Retention

All 11 features confirmed by the Boruta algorithm were directly entered into a multivariable logistic regression model to construct the final predictive nomogram, without any post-hoc *p*-value based stepwise removal. Although certain retained variables did not reach traditional statistical significance (*p* < 0.05) in the final multivariable regression, we deliberately kept them in the model. This decision is supported by two main reasons: first, Boruta relies on ensemble tree structures that capture non-linear relationships and feature interactions which linear models often miss; second, these predictors possess strong biological plausibility in aortic surgery. To assess predictor-selection stability, the Boruta algorithm was re-run independently within each of 1000 bootstrap samples, applied to the 40 variables that had passed the VIF filter in the original analysis. The proportion of bootstrap samples in which each variable was selected (ranking ≤ 2) was recorded.

#### 2.5.3. Model Validation and Performance

The discriminatory performance of the model was assessed using the area under the receiver operating characteristic (ROC) curve (AUC). Model calibration was evaluated through calibration plots alongside advanced quantitative metrics, specifically calibration-in-the-large and calibration slope, rather than relying heavily on the Hosmer–Lemeshow goodness-of-fit test. The clinical utility of the model was determined by decision curve analysis (DCA), which quantified the net benefit across a range of clinically relevant risk thresholds. Finally, the robustness of the model was internally validated using 1000 bootstrap resamples, allowing for the calculation of optimism-corrected performance estimates to minimize potential overfitting. To facilitate bedside interpretation, model predictions were translated into absolute risks of POD for each individual rather than being reported solely as odds ratios. The accompanying web-based calculator directly outputs the estimated absolute probability of POD for a given set of predictor values, thereby converting the multivariable logistic regression coefficients into clinically meaningful, patient-specific risk estimates. Prediction model risk of bias assessment tool (PROBAST) was used to assess risk of bias in model development and internal validation.PROBAST for artificial intelligence (PROBAST-AI) was not applicable as the final model is a logistic regression nomogram.

### 2.6. Statistical Analysis

Distributed data are presented as mean ± standard deviation (SD) and compared using the Welch two-sample *t*-test. Non-normally distributed data are presented as median (interquartile range, Q1–Q3) and compared using the Wilcoxon rank-sum test. Categorical variables are presented as frequencies (percentages) and compared using Pearson’s Chi-squared test or Fisher’s exact test. A complete-case analysis was utilized, and potential selection bias was addressed by comparing baseline characteristics between included and excluded patients. Due to marked right-skewness, all continuous inflammatory biomarkers and ratios were natural log-transformed (ln(x+1)) prior to regression modeling.

## 3. Results

### 3.1. Study Population and Baseline Characteristics

A total of 355 patients who underwent surgical repair for ATAAD were included in this study. Among the cohort, 106 patients (29.86%) developed POD. As illustrated in the histogram (Figure S1), the initial onset of POD occurred well beyond the 6-h blood sampling window (median onset time: 41.6 h), confirming that our blood biomarkers were captured at a pre-onset baseline rather than during established delirium. Table 1 summarizes the baseline demographics, comorbidities, and extent of anatomical involvement of the study population. Patients who developed POD were significantly older than those who remained delirium-free (58 ± 11 vs. 53 ± 11 years, *p* < 0.001, SMD = 0.48). Furthermore, patients in the delirium group presented with higher baseline EuroSCORE I values compared to the non-delirium group (median 9.0 (8.0, 11.0) vs. 8.0 (8.0, 10.0), *p* < 0.001, SMD = 0.53). Other baseline characteristics, including sex, BMI, and the prevalence of comorbidities such as hypertension, type 2 diabetes mellitus, coronary artery disease, and stroke, did not show statistically significant differences between the two groups (*p* > 0.05). Similarly, the symptom-to-surgery interval was comparable between the delirium and non-delirium groups (20 (11, 3])vs. 20 (11, 35) hours, *p* = 0.976). Regarding the anatomical extent of dissection, including the involvement of the brachiocephalic trunk, subclavian and carotid arteries, and visceral or renal arteries, no significant disparities were observed between the two groups (all *p* > 0.05). These findings suggest that advanced age and a higher preoperative surgical risk, as quantified by the EuroSCORE I, are the primary baseline clinical factors associated with the development of POD following ATAAD surgery.

### 3.2. Operative and Postoperative Characteristics

Regarding intraoperative factors, no significant differences were observed in CPB time, aortic cross-clamp (ACC) time, lowest nasopharyngeal temperature, or the volume of intraoperative blood product transfusions (RBCs and FFP) between the delirium and non-delirium groups (*p* > 0.05, Table 2). However, patients who developed POD exhibited significantly worse clinical outcomes (Table 2). Specifically, these patients had higher sequential organ failure assessment (SOFA) scores on postoperative days 1 and 2 (*p* < 0.001). Furthermore, the delirium group required significantly longer mechanical ventilation (median 52 vs. 20 h, *p* < 0.001) and had longer ICU and hospital lengths of stay compared to their delirium-free counterparts (*p* < 0.001). While in-hospital mortality was numerically higher in the delirium group, this difference did not reach statistical significance (8.5% vs. 5.6%, *p* = 0.315).

### 3.3. Exploratory Evaluation of Perioperative Inflammatory Biomarkers Across Time Points

**Figure 2:**
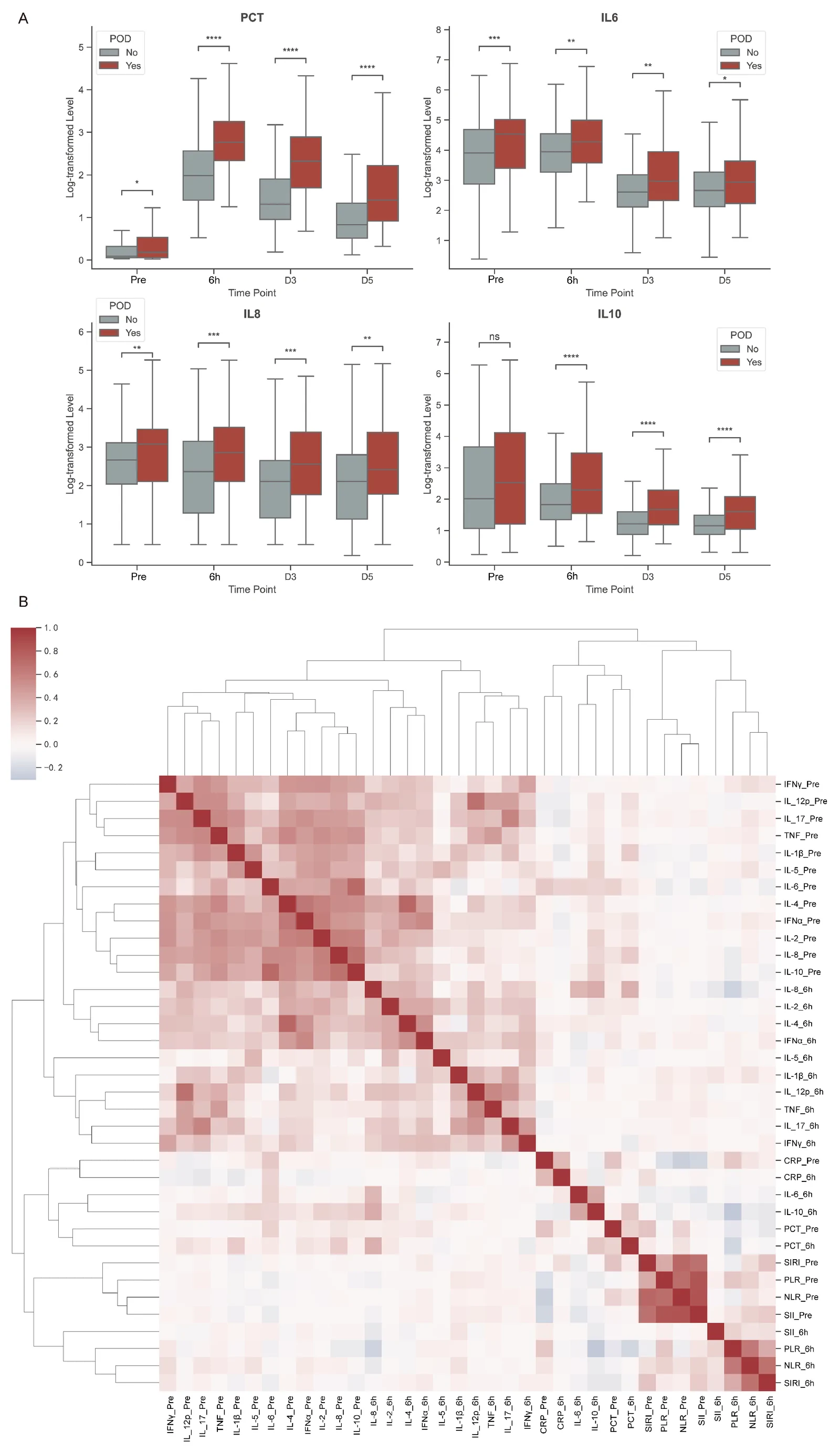
(**A**) Serum PCT, IL−6, IL−8, IL−10 levels in ATAAD patients with/without POD across four time points (Preop–D5). Box plots show median (IQR); *p* by Mann−Whitney U. Significance: *—*p* < 0.05, **—*p* < 0.01, ***—*p* < 0.001, ****—*p* < 0.0001; (**B**) Correlation heatmap with hierarchical clustering among biomarkers and clinical variables. Color scale: −1.0 (blue) to +1.0 (red); *p* < 0.05.

To provide a descriptive overview of the broader systemic response, serial inflammatory measurements were evaluated across multiple postoperative time points (Figure 2A). At several individual postoperative time points, patients who developed POD had higher levels of PCT, IL-6, IL-8, and IL-10 compared to the non-delirium group (*p* < 0.05 at the corresponding time points; Table S1). These comparisons were exploratory and cross-sectional at each time point, and no formal longitudinal group-by-time interaction testing was performed. We also analyzed the correlation among perioperative inflammatory markers and hematological indices. Although we monitored these markers up to postoperative day 5 to capture the broader systemic response, we deliberately focused our subsequent predictive model development exclusively on preoperative and early postoperative (6h) markers. This strategic restriction was driven by the critical clinical urgency for early bedside risk stratification, which provides clinicians with a narrow yet vital window to implement targeted preventive strategies during the acute phase of ATAAD repair. As depicted in the hierarchical clustering heatmap (Figure 2B), the pairwise correlation analysis of perioperative inflammatory and hematological variables revealed potential clustering and correlation patterns, reflecting the complexity of the systemic response during surgical recovery.

### 3.4. Variable Selection and Model Development

**Figure 3:**
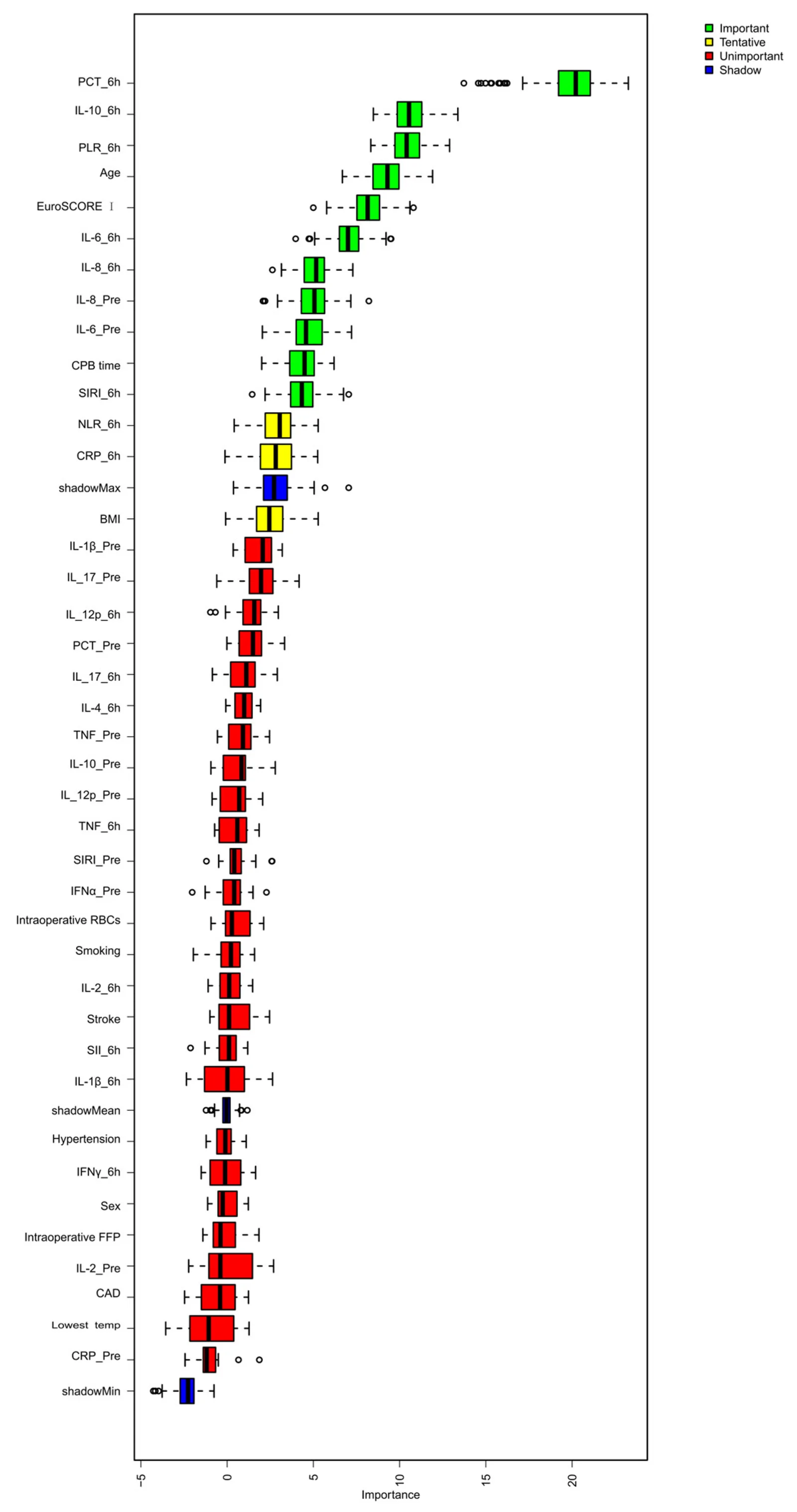
Feature selection and variable importance evaluated by the Boruta algorithm. Box plots display the importance scores of candidate predictors across random forest iterations. Green, yellow, and red box plots indicate “Confirmed” (Important), “Tentative,” and “Rejected” (Unimportant) variables, respectively, with blue box plots representing shadow attributes.

To minimize potential multicollinearity among the candidate predictors, we performed a two-stage variable selection process. First, VIF was calculated for all 48 initial variables, including preoperative comorbidities, intraoperative factors, and serial inflammatory markers. As shown in Figure S2, 8 variables with a VIF exceeding 5 were removed to limit multicollinearity. Next, the remaining 40 variables were evaluated using the Boruta feature selection algorithm. The algorithm classified 11 variables as important, 26 as unimportant, and 3 as tentative (Figure 3). The 11 confirmed predictors were SIRI_6h, PLR_6h, IL−10_6h, IL−8_6h, IL−8_Pre, IL−6_6h, IL−6_Pre, PCT_6h, EuroSCORE I, CPB time, and age. The 26 rejected variables comprised SIRI_pre, SII_6h, TNF_6h, TNF_Pre, IFN−γ_6h, IFN−γ_Pre, IL−17_6h, IL−17_Pre, IL−12p_6h, IL−12p_Pre, IL−10_Pre, IL−4_6h, IL−2_6h, IL−2_Pre, IL−1β_D1, IL−1β_Pre, PCT_Pre, CRP_Pre, intraoperative plasma transfusion, intraoperative red blood cell transfusion, lowest nasopharyngeal temperature, history of stroke, history of coronary heart disease, history of hypertension, smoking history, and gender. NLR_6h, CRP_6h, and BMI remained tentative. This procedure allowed the final model to incorporate both inflammatory profiles and clinical baseline factors.

Multivariable logistic regression analysis was performed to evaluate the independent associations of the 11 variables identified by the Boruta algorithm with POD. The regression parameters, including adjusted odds ratios (ORs) and 95% confidence intervals (CIs), are detailed in Table S2. While certain factors, such as PCT_6h (OR = 3.65; 95% CI: 2.44—5.66; *p* < 0.001) and IL−6_6h (OR = 1.69; 95% CI: 1.25—2.34; *p* < 0.001), showed strong independent associations with POD, several other candidate predictors did not reach statistical significance (*p* < 0.05) within the multivariable model. Despite these non-significant *p* values, we retained these clinically recognized variables (e.g., age, EuroSCORE I, and CPB time) and specific inflammatory markers (e.g., PLR_6h, IL−8_6h) in the final model, given their high importance scores in the Boruta analysis and their prior documentation in the literature regarding post-surgical neurological outcomes. To facilitate manual computation and clinical application, the logit equation used for the online risk calculator is expressed as follows: Logit(P) = −7.0945 + 0.0326 × (Age) − 0.6280 × (SIRI_6h) − 0.3549 × (PLR_6h) − 0.0354 × (IL−10_6h) − 0.2176 × (IL−8_6h) + 0.0541 × (IL−8_pre) + 0.5267 × (IL−6_6h) + 0.2084 ×(IL−6_pre) + 1.2957 × (PCT_6h) + 0.1667 × (EuroSCORE I) + 0.0041 × (CPB time).

### 3.5. Development of the Predictive Nomogram and Model Performance

**Figure 4:**
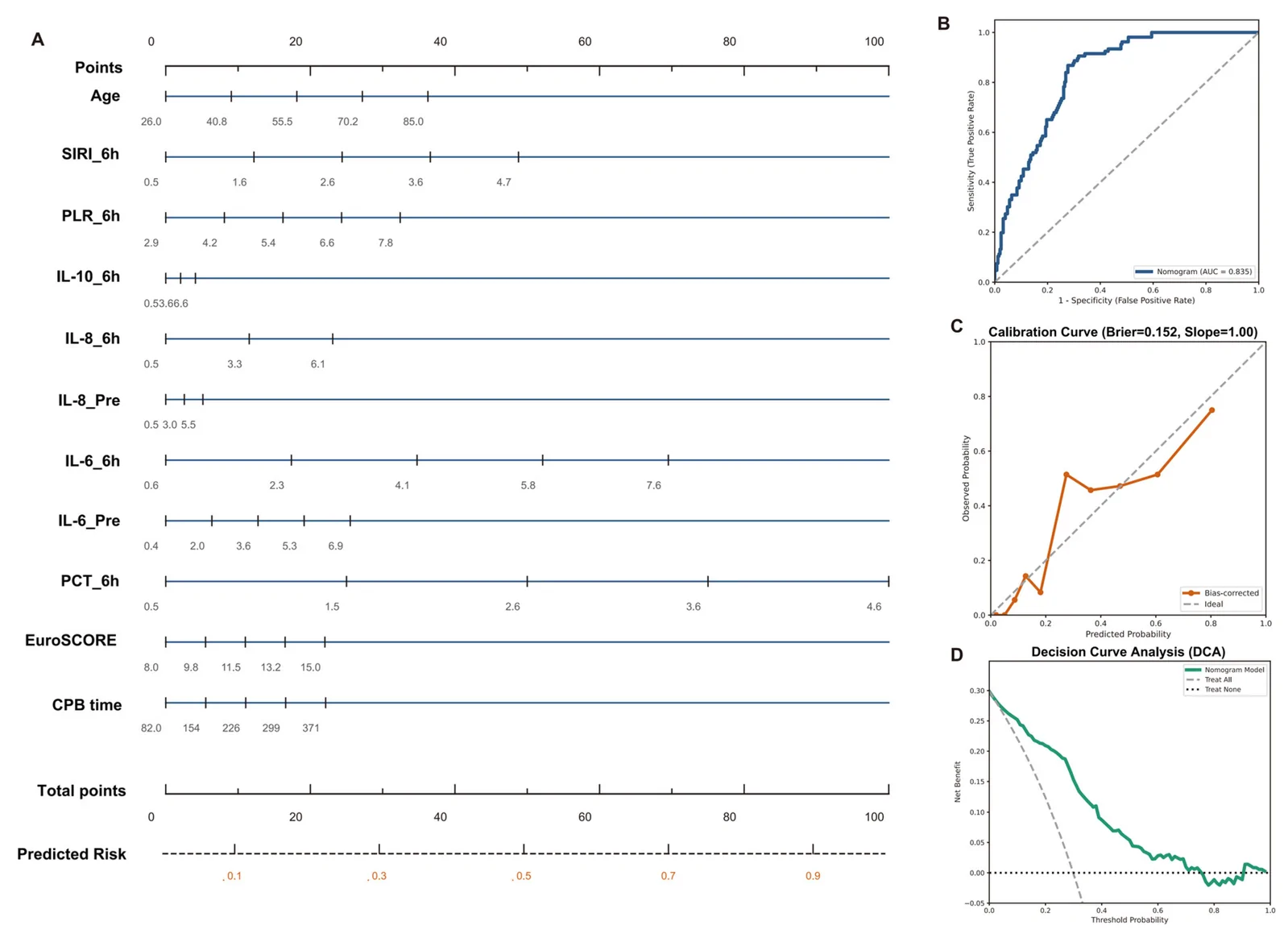
Performance evaluation of the predictive nomogram. (**A**) The predictive nomogram incorporating the 11 Boruta−selected predictors (Age, EuroSCORE I, CPB time, SIRI_6h, PLR_6h, IL−10_6h, IL−8_6h, IL−8_pre, IL−6_6h, IL−6_pre, PCT_6h) for estimating the risk of POD following ATAAD repair. (**B**) ROC curve demonstrating the discriminatory performance of the model (AUC = 0.835). (**C**) Calibration curve evaluating the agreement between predicted risks and observed outcomes (Brier score = 0.152, calibration slope = 1.00). (**D**) DCA illustrating the clinical net benefit across a range of threshold probabilities compared to “treat-all” and “treat-none” strategies.

To facilitate individual risk estimation, a predictive nomogram was constructed by integrating the 11 identified predictors (Figure 4A). In this model, each predictor corresponds to a specific point on the top “Points” axis based on its value. By summing the points assigned to each clinical and inflammatory variable, derive a “Total Points” sum that correlates with the estimated probability of POD following ATAAD repair. As shown in Figure 4A, variables such as PCT_6h and IL−6_6h exert a substantial influence on the total score, while baseline factors like age and EuroSCORE I provide necessary clinical context for individual assessment.

The discriminatory performance of the nomogram was evaluated using ROC curve analysis. As shown in Figure 4B, the model achieved an AUC of 0.835 (95% CI: 0.794–0.876), indicating reasonable internal discrimination in identifying patients at high risk of POD following ATAAD repair. This performance suggests that integrating acute inflammatory markers (e.g., PCT_6h, IL−6_6h) and established clinical risk factors captures complementary aspects of patient vulnerability. While encouraging, these results reflect internal cohort performance and require external validation across independent cohorts prior to any clinical consideration.

To assess model calibration, quantitative calibration metrics were evaluated alongside bias-corrected calibration plots (Figure 4C). The model demonstrated good calibration performance, yielding a calibration in the large of −0.0000 and a calibration slope of 1.0000, suggesting a close agreement between predicted and observed risks with minimal systematic miscalibration. The DCA curve demonstrated a positive net benefit over “treat-all” or “treat-none” strategies across threshold probabilities ranging from approximately 0 to 0.7 (Figure 4D), suggesting a net clinical benefit under exploratory evaluation. To support transparency and further research, an exploratory web-based risk calculator was implemented (https://adream20100-debug.github.io/ataad-pod-calculator/, accessed on 28 August 2026). This interactive tool allows users to input patient data to obtain a real-time risk estimate, serving as a preliminary resource for future independent testing.

To evaluate the clinical applicability and reclassification performance of the nomogram, we assessed its performance across a range of clinically justified decision thresholds (15%, 20%, and 25%) (Table S3). At a threshold of 15%, the model achieved a high sensitivity of 93.4% and a negative predictive value (NPV) of 95.2%, classifying 208 patients (58.6%) as high risk. As the threshold increased to 25%, the specificity improved to 69.5% with a positive predictive value (PPV) of 55.3%, identifying 170 high-risk patients (47.9%). These metrics provide clinicians with a flexible framework to tailor interventions based on institutional risk tolerance and resource availability.

### 3.6. Model Robustness and Sensitivity Analyses

Several sensitivity analyses were performed to test the stability and reliability of the model. Because 108 of the 463 screened patients (23.3%) were excluded due to missing data (with preoperative IL−6 and IL−8 showing missing rates of 11.02%, and other variables ranging from 0.00% to 1.08%), we evaluated potential selection bias. As detailed in Supplementary Table S4, a comparison between included and excluded patients showed no significant differences in the primary endpoint of POD (29.9% vs. 25.0%, *p* = 0.328) or in-hospital mortality (6.5% vs. 8.3%, *p* = 0.506). However, several baseline and anatomical characteristics (including coronary artery disease and anatomical involvement variables) and ICU length of stay differed significantly between the two groups, indicating that the excluded patients were not fully comparable to the included cohort. A multiple-imputation sensitivity analysis (m = 20 imputed datasets; Rubin’s rules for combining estimates) yielded an apparent AUC of 0.812 (95% CI 0.803–0.821) and an optimism-corrected AUC of 0.786 after 200 Bootstrap resamples (Figure S3), suggesting that the complete-case results were not materially altered by the missing-data handling, although selection bias due to missing data cannot be fully excluded. Additionally, to address the unexpected inverse association observed for SIRI_6h (unadjusted OR: 0.547, 95% CI: 0.395–0.757, *p* < 0.0001; adjusted OR: 0.645, 95% CI: 0.429–0.968, *p* = 0.0344), we performed additional sensitivity analyses to test for potential multicollinearity or model instability among leukocyte-derived ratios. As shown in Supplementary Table S5, omitting PLR_6h (OR: 0.571, *p* = 0.0038) or restricting the model exclusively to hematological and clinical baseline variables (OR: 0.617, *p* = 0.0154) yielded virtually unchanged directions and significance, confirming the robustness of this finding. Finally, we tested for potential non-linear relationships using restricted cubic splines (RCSs). Although exploratory RCS modeling captured complex trajectories (Figure S4), full non-linear transformations offered only a marginal change in overall discrimination compared to the linear model (AUC: 0.835 vs. 0.855) (Figure S5). Because complex splines would reduce the practical usability and bedside accessibility of a printed nomogram, a linear specification was retained for clinical use. Finally, a 1000-resample internal bootstrap validation was conducted to account for selection-induced optimism, resulting in a robust optimism-corrected AUC of 0.811 (mean optimism, 0.0247) (Figure S5). These supplemental evaluations support the internal consistency of the selected modeling approach while acknowledging the need for subsequent external validation.

## 4. Discussion

Our study indicates that POD after total arch replacement for ATAAD is preceded by an acute inflammatory surge, and that integrating early inflammatory markers with established clinical variables improves risk stratification. This interpretation aligns with broader evidence that postoperative inflammation is closely linked to POD across surgical populations, with elevated IL−6, IL−8 and CRP repeatedly observed in patients who develop delirium [14,17]. Cardiac surgery appears especially relevant to this mechanism because CPB induces a systemic inflammatory response, and this postoperative inflammatory burden has been repeatedly associated with neurologic and other adverse outcomes [19,20,21]. The identification of postoperative IL−6 as an independent predictor of POD is well aligned with existing evidence. Meta-analytic data show that IL−6 is consistently higher in POD patients, and cardiac surgery cohorts report that postoperative IL−6 rises further in patients who develop POD or postoperative cognitive dysfunction [14,17,21,22]. IL−6 is also biologically plausible as a mediator rather than a passive correlate, because inflammatory markers can compromise BBB permeability, and neuroinflammation is increasingly recognized as a central pathway in POD pathogenesis [10,14,23]. The signal for PCT in our cohort is likewise credible, as postoperative PCT independently predicts POD in ICU patients and persistent postoperative elevations of PCT and IL−6 have been linked to worse complications after cardiac surgery [20,21]. A notable finding in our multivariable model (Table S2) was the inverse association between SIRI_6h and POD risk (adjusted OR = 0.53, *p* = 0.011). However, this inverse association requires careful interpretation and should not be construed as a direct biological protective effect. From a statistical perspective, this phenomenon is likely driven by multivariable adjustment and potential suppression effects within our high-dimensional model. Specifically, when dominant, potent acute-phase drivers of delirium (such as postoperative IL−6 and PCT) are simultaneously controlled for, the residual variance of SIRI_6h may capture distinct immune-regulatory balances or relative lymphocyte preservation rather than pure tissue injury. Although overall multicollinearity was controlled after removing 8 variables with a VIF > 5, structural overlap and shared mathematical components among composite leukocyte ratios in multivariable regression models can occasionally lead to directional shifts compared to unadjusted observations. This contrasts with simpler single ratios and the literature on postoperative NLR in cardiac surgery, where higher values generally track with adverse neurological outcomes. However, as demonstrated across various cardiovascular settings, composite indices like SIRI often exhibit heterogeneous prognostic behavior, reflecting a broader, complex interplay between innate inflammatory activation and adaptive immune competence rather than injury burden alone [14,24,25]. While NLR, PLR, SII, and SIRI remain accessible and low-cost markers, their standalone performance and directional associations vary notably by outcome and clinical population [25,26].

Our mechanistic interpretation supports convergence between systemic inflammation, BBB dysfunction, and neuroglial activation. Reviews and translational studies indicate that surgery-induced inflammation disrupts endothelial integrity and BBB function, allowing peripheral inflammatory mediators to amplify central neuroinflammation [23]. Experimental models further show that surgery can increase BBB permeability, activate microglia, and produce delirium-like behavior, while BBB-protective interventions attenuate these changes [27,28]. These data support the view that the immediate postoperative inflammatory burst is more clinically relevant than baseline inflammation alone in triggering POD [20,21,23]. Metabolic and neuronal vulnerability likely interact with this inflammatory cascade. Contemporary mechanistic reviews describe POD as a multifactorial syndrome involving cytokine-mediated microglial activation, neurotransmitter dysregulation, oxidative stress, and synaptic dysfunction [9,10,11]. Biomarker studies also link POD with indicators of axonal and glial injury, including NFL, GFAP, tau-related markers, and p-tau181, which suggests that systemic inflammation may couple with structural brain vulnerability in susceptible patients [17,22]. In ATAAD, this interaction is plausibly intensified by the hyperacute disease state, urgent repair, and CPB-related inflammatory stress [5,6].

From a clinical standpoint, our nomogram offers an exploratory framework that compares favorably with existing models, especially because it uses readily available early postoperative markers rather than delayed or highly specialized testing. Prior nomogram studies consistently show that POD models can provide useful discrimination and favorable decision-curve performance, but most were developed in mixed cardiac populations rather than ATAAD-specific cohorts [19,29]. ATAAD and related aortic-dissection populations likely require tailored tools, because their inflammatory and hemodynamic profiles differ from routine cardiac surgery [29,30]. Patients identified as high-risk by our 11-variable model are intended to trigger a bundled, multi-component non-pharmacological and targeted pharmacological delirium prevention protocol. This includes early mobilization, minimizing deep sedation or avoiding specific high-risk sedatives (e.g., benzodiazepines), optimization of sleep–wake cycles, sensory reorientation (ensuring glasses and hearing aids are used), and early interdisciplinary consultation. By defining these proactive measures, the net benefit calculated via DCA appropriately weighs the clinical advantages of preventing POD against the minor harms, costs, and resource allocation associated with these heightened monitoring and preventive interventions.

To assess the stability of the variable-selection procedure, we performed a separate bootstrap stability analysis in which the Boruta algorithm was re-run independently within each of the 1000 bootstrap samples, applied to the 40 variables that had passed the VIF filter in the original analysis. All 11 final predictors were selected in ≥91% of bootstrap samples (PLR_6h, IL−10_6h, and PCT_6h: 100%; IL−6_6h: 99.7%; age and IL−8_6h: 99.4%; SIRI_6h: 99.0%; IL−8_Pre: 98.8%; IL−6_Pre: 96.6%; CPB time: 95.4%; EuroSCORE I: 91.0%), indicating that their selection is highly stable. A full table of selection proportions for all 40 candidate variables is provided in Supplementary Table S6. We note that several additional variables were also selected in a high proportion of bootstrap samples (e.g., NLR_6h: 95.4%, BMI: 92.8%, CRP_6h: 89.6%) but were not retained in the final 11-variable model. This is not unexpected. Boruta is based on random forests, which are relatively insensitive to collinearity; when multiple correlated variables carry similar predictive information, Boruta may select any of them across different bootstrap samples. In the present analysis, NLR_6h, BMI, and CRP_6h are correlated with the final predictors (e.g., NLR_6h with SIRI_6h and PLR_6h), and their high selection proportions therefore reflect shared inflammatory information rather than independent predictive value. This phenomenon is a well-recognized limitation of feature-selection algorithms in the presence of collinearity and does not affect the stability of the final 11-variable model.

Several limitations warrant consideration. First, as a retrospective, single-center study, our findings require external validation in larger, multicenter prospective cohorts to confirm generalizability and transportability, and the absence of a formal a priori sample size calculation should be considered a study constraint. Second, potential biases must be acknowledged. The use of complete-case analysis raises the possibility of selection bias, given that 108 of the 463 screened patients (23.3%) were excluded due to missing data. Although the incidence of POD did not differ significantly between included and excluded patients, several baseline and anatomical characteristics and ICU length of stay differed significantly between the two groups (Supplementary Table S4). Therefore, selection bias cannot be excluded, and the multiple-imputation sensitivity analysis should be interpreted as providing supportive, rather than definitive, evidence. Additionally, information bias may have arisen from the twice-daily CAM-ICU assessment schedule, although trained ICU physicians performed all assessments to ensure consistency. Third, unmeasured confounders (such as pre-existing cognitive impairment, specific perioperative sedation practices, and detailed delirium screening frequency) may have influenced our findings. Finally, with 355 patients and 106 POD events, the overall events-per-variable (EPV) ratio was approximately 9.6 (106 events/11 predictors). While this exceeds the conventional minimum threshold of EPV ≥ 10 narrowly and falls below the more conservative EPV ≥ 20 recommendation for penalised regression, it is broadly consistent with EPV ≥ 5−10 benchmarks cited in the prediction modelling literature. Nevertheless, the relatively modest EPV introduces a non-negligible risk of model overfitting and optimism in performance estimates, as formally recognised by PROBAST Domain 4 (Supplementary Table S7a,b), which assigns a high risk of bias rating when EPV < 10. Although we applied several mitigation strategies—including variance inflation factor screening (VIF < 5), the Boruta ensemble feature selection algorithm to reduce predictor dimensionality, and 1000-resample bootstrap internal validation with optimism correction (yielding an optimism-corrected C-index of 0.811)—these measures cannot fully eliminate the uncertainty introduced by limited event counts. Future research should prioritize investigating the dynamic trajectories of these biomarkers over the first 72 h and utilizing machine learning algorithms to integrate longitudinal data, which may further enhance predictive accuracy and reveal deeper mechanistic insights through mediation analysis.

In conclusion, this study establishes a promising, exploratory nomogram for POD risk assessment in ATAAD patients. By integrating early postoperative inflammatory markers with established clinical variables, this preliminary model provides a robust foundation for early risk stratification, though rigorous multicenter external validation is required before real-world clinical implementation can be considered.

## Figures and Tables

**Table 1:** Patient demographics and baseline characteristics. Values are presented as mean ± standard deviation (SD) for normally distributed continuous variables, median (interquartile range (IQR) for non-normally distributed continuous variables, and *n* (%) for categorical variables. *P*-values were calculated using the following tests (superscript numbers indicate the test applied to each variable): ^1^ Welch’s two-sample *t*-test; ^2^ Wilcoxon rank sum test; ^3^ Pearson’s chi-squared test; ^4^ Fisher’s exact test. SMD, standardized mean difference (|SMD| < 0.1 indicates negligible between-group imbalance). Abbreviations: BMI, body mass index; T2DM, type 2 diabetes mellitus; CAD, coronary artery disease; SD, standard deviation; IQR, interquartile range; SMD, standardized mean difference.

Characteristic	Overall (*n* = 355)	No Delirium (*n* = 249)	Delirium (*n* = 106)	*p*-Value	SMD
**Demographics**					
**Age, years**	55 ± 11	53 ± 11	58 ± 11	<0.001 ^1^	−0.48
**Sex (male), *n* (%)**	266 (74.9%)	188 (75.5%)	78 (73.6%)	0.703 ^3^	0.04
**BMI, kg/m^2^**	24.8 (22.9, 27.1)	25.1 (23.1, 27.0)	24.5 (22.1, 27.1)	0.115 ^2^	0.22
**Comorbidities**					
**Smoking, *n* (%)**	173 (48.7%)	116 (46.6%)	57 (53.8%)	0.215 ^3^	0.14
**Hypertension, *n* (%)**	232 (65.4%)	166 (66.7%)	66 (62.3%)	0.425 ^3^	0.09
**T2DM, *n* (%)**	9 (2.5%)	7 (2.8%)	2 (1.9%)	>0.999 ^4^	0.06
**CAD, *n* (%)**	11 (3.1%)	8 (3.2%)	3 (2.8%)	>0.999 ^4^	0.02
**Stroke, *n* (%)**	15 (4.2%)	8 (3.2%)	7 (6.6%)	0.157 ^4^	0.16
**Symptom-to-surgery interval, hours**	20 (11, 36)	20 (11, 35)	20 (11, 36)	0.976 ^2^	0.08
**EuroSCORE I**	8.00 (8.00, 10.00)	8.00 (8.00, 10.00)	9.00 (8.00, 11.00)	<0.001 ^2^	−0.53
**Extent of anatomical involvement**					
**Brachiocephalic trunk, *n* (%)**	240 (67.6%)	164 (65.9%)	76 (71.7%)	0.282 ^3^	0.13
**Left subclavian artery, *n* (%)**	255 (71.8%)	181 (72.7%)	74 (69.8%)	0.581 ^3^	0.06
**Left common carotid artery, *n* (%)**	230 (64.8%)	162 (65.1%)	68 (64.2%)	0.870 ^3^	0.02
**Celiac trunk, *n* (%)**	86 (24.2%)	64 (25.7%)	22 (20.8%)	0.319 ^3^	0.12
**Superior mesenteric artery, *n* (%)**	55 (15.5%)	41 (16.5%)	14 (13.2%)	0.437 ^3^	0.09
**Inferior mesenteric artery, *n* (%)**	39 (11.0%)	30 (12.0%)	9 (8.5%)	0.327 ^3^	0.12
**Unilateral renal artery, *n* (%)**	140 (39.4%)	102 (41.0%)	38 (35.8%)	0.367 ^3^	0.11
**Bilateral renal artery, *n* (%)**	21 (5.9%)	13 (5.2%)	8 (7.5%)	0.395 ^3^	0.10
**Coronary artery, *n* (%)**	31 (8.7%)	22 (8.8%)	9 (8.5%)	0.916 ^3^	0.01

**Table 2:** Patient operative factors and postoperative outcomes. Continuous variables are presented as median (interquartile range (IQR); categorical variables as *n* (%). *P*-values were calculated using the following tests (superscript numbers indicate the test applied to each variable): ^1^ Wilcoxon rank sum test; ^2^ Pearson’s Chi-squared test; SMD, standardized mean difference. |SMD| < 0.1 indicates no meaningful difference between groups. Abbreviations: CPB, cardiopulmonary bypass; ACC, aortic cross-clamp; RBCs, red blood cells; FFP, fresh frozen plasma; SOFA, sequential organ failure assessment; ICU, intensive care unit; LOS, length of stay; IQR, interquartile range; SMD, standardized mean difference.

Characteristic	Overall (*n* = 355)	No Delirium (*n* = 249)	Delirium (*n* = 106)	*p*-Value	SMD
**Operative Factors**					
**CPB time, min**	152 (135, 179)	151 (135, 173)	162 (135, 191)	0.088 ^1^	−0.31
**ACC time, min**	89 (75, 106)	88 (75, 102)	90 (76, 114)	0.264 ^1^	−0.16
**Lowest nasopharyngeal temp, °C**	27.70 (26.50, 28.20)	27.50 (26.50, 28.20)	28.00 (26.50, 28.20)	0.622 ^1^	−0.05
**Intraoperative RBCs, U**	4.00 (2.00, 6.00)	4.00 (2.00, 6.00)	4.00 (2.00, 6.00)	0.180 ^1^	−0.15
**Intraoperative FFP, mL**	400 (400, 600)	400 (400, 600)	400 (400, 600)	0.290 ^1^	−0.05
**Postoperative Outcomes**					
**SOFA score (d** **1** **)**	7.00 (6.00, 8.00)	7.00 (5.00, 8.00)	8.00 (7.00, 9.00)	<0.001 ^1^	−0.71
**SOFA score (d2)**	7.00 (6.00, 8.45)	6.96 (6.00, 8.00)	8.68 (7.00, 10.00)	<0.001 ^1^	−1.02
**SOFA score (d** **3** **)**	7.00 (5.78, 8.00)	6.23 (5.00, 7.08)	8.00 (6.63, 10.38)	<0.001 ^1^	−0.4
**Mechanical ventilation, h**	26 (13, 57)	20 (11, 37)	52 (26, 109)	<0.001 ^1^	−0.5
**ICU LOS, d**	5 (4, 8)	4 (3, 6)	8 (5, 15)	<0.001 ^1^	−0.68
**Hospital LOS, d**	17 (14, 21)	16 (14, 19)	19 (16, 28)	<0.001 ^1^	−0.58
**In-hospital mortality, *n* (%)**	23 (6.5%)	14 (5.6%)	9 (8.5%)	0.315 ^2^	0.11

## Data Availability

The datasets generated and/or analyzed during the current study are not publicly available due to patient privacy regulations but are available from the corresponding author upon reasonable request.

## Supplementary Materials

The following supporting information can be downloaded at: https://www.mdpi.com/article/10.3390/jcm15208090/s1, Figure S1: Time-to-Onset Distribution of POD. The histogram illustrates the temporal distribution of initial POD onset among all patients who developed delirium (n = 106). The vertical dashed line indicates the 6-h postoperative time point at which all predictive biomarker samples were collected (mean onset: 50.9 h; median onset: 41.6 h). Figure S2: VIF analysis of predictors in ATAAD-POD model (log_10_ scale). Green diamonds = VIF < 5 (acceptable); red zone = VIF ≥ 5 (high collinearity). All VIF < 5 variables retained. See Table 1 for variable definitions. Figure S3: ROC curve with bootstrap internal validation for the multiple-imputation sensitivity analysis model. The apparent AUC is 0.812, and the optimism-corrected AUC yielded by the multiple-imputation sensitivity analysis is 0.786. Figure S4: RCS curves capturing the complex non-linear trajectories between continuous predictors and predicted probability. Exploratory RCS modeling illustrates the adjusted associations and non-linear shapes for individual predictors, including Age, SIRI_6h, PLR_6h, IL-10_6h, IL-8_6h, IL-8_Pre, IL-6_6h, IL-6_Pre, PCT_6h, EuroSCORE, and CPB Time. The y-axis represents the predicted probability, and the x-axis represents the respective predictor values. Figure S5: ROC curve comparison between the linear model and the RCS model. The blue solid line represents the linear model (apparent AUC = 0.835, bias-corrected AUC = 0.811 following a 1000-resample internal bootstrap validation), while the orange dashed line represents the full non-linear RCS model (apparent AUC = 0.855, bias-corrected AUC = 0.809). Table S1: Perioperative Inflammatory Markers and Hematological Indices. Continuous variables are presented as median (interquartile range [IQR]). *p*-values were calculated using the following tests (superscript numbers indicate the test applied to each variable): ^1^ Welch Two Sample *t*-test; ^2^ Wilcoxon rank sum test; ^3^ Pearson’s Chi-squared test; ^4^ Fisher’s exact test.Abbreviations: CRP, C-reactive protein; PCT, procalcitonin; IL, interleukin; IFN, interferon; TNF, tumor necrosis factor; NLR, neutrophil-to-lymphocyte ratio; PLR, platelet-to-lymphocyte ratio; SII, systemic immune-inflammation index; SIRI, systemic inflammation response index; Pre-op, preoperative; Post-op, postoperative; IQR, interquartile range. Table S2: Multivariable Logistic Regression Analysis for POD. Table S3: Clinical Utility Across Decision Thresholds. Table S4: Comprehensive Comparison of Baseline Characteristics, Intraoperative Factors, and Postoperative Outcomes Between Included Patients and Patients Excluded Due to Missing Data. Table S5: Association Between SIRI_6h and POD Across Various Model Specifications. Table S6: A full table of selection proportions for all 40 candidate variables. Table S7a: PROBAST Domain-Level Assessment. Table S7b: PROBAST Step-Level Summary. TRIPOD Checklist.

## References

[1] Zhu Y., Lingala B., Baiocchi M., Tao J.J., Arana V.T., Khoo J.W., Williams K.M., Traboulsi A.A.-R., Hammond H.C., Lee A.M. (2020). Type A Aortic Dissection-Experience Over 5 Decades: JACC Historical Breakthroughs in Perspective. J. Am. Coll. Cardiol..

[2] Ma L., Chai T., Yang X., Zhuang X., Wu Q., Chen L., Qiu Z. (2022). Outcomes of hemi- vs. total arch replacement in acute type A aortic dissection: A systematic review and meta-analysis. Front. Cardiovasc. Med..

[3] Iino K., Takago S., Saito N., Ueda H., Yamamoto Y., Kato H., Kimura K., Takemura H. (2022). Total arch replacement and frozen elephant trunk for acute type A aortic dissection. J. Thorac. Cardiovasc. Surg..

[4] Lerdpunnapongse P., Slisatkorn W., Wongkornrat W., Sanphasitvong V. (2023). Comparison of the outcomes between total arch replacement and nontotal arch replacement in patients with acute type A aortic dissection. Indian J. Thorac. Cardiovasc. Surg..

[5] Okada K., Kotani S., Ozawa K., Kishinami G., Yamamoto A., Cho Y. (2024). Cerebral Protection with Deep Hypothermic Circulatory Arrest During Total Arch Replacement Using the Arch-First Technique for Acute Aortic Dissection. Cureus.

[6] Ravesh M.S., Friedrich C., Schoettler J., Hummitzsch L., Elke G., Salem M., Lutter G., Puehler T., Cremer J., Haneya A. (2022). Association between Duration of Deep Hypothermic Circulatory Arrest and Surgical Outcome in Patients with Acute Type A Aortic Dissection: A Large Retrospective Cohort Study. J. Clin. Med..

[7] Lin J.L., Zheng G.Z., Chen L.W., Luo Z.R. (2023). A nomogram model for assessing predictors and prognosis of postoperative delirium in patients receiving acute type A aortic dissection surgery. BMC Cardiovasc. Disord..

[8] Liu C., Zhang L., Tang W., Zhao S., Li M., Li J., Shao Y. (2024). Serum NPTX2 as a Potential Predictive Biomarker for Postoperative Delirium in Patients with Acute Type A Aortic Dissection. Neuropsychiatr. Dis. Treat..

[9] Leng K., Maze M., Barreto Chang O.L. (2025). Emerging biomarkers of postoperative delirium at the intersection of neuroinflammation and neurodegeneration. Front. Aging Neurosci..

[10] Xiao M.Z., Liu C.X., Zhou L.G., Yang Y., Wang Y. (2023). Postoperative delirium, neuroinflammation, and influencing factors of postoperative delirium: A review. Medicine.

[11] Koh H.J., Joo J. (2025). The Role of Cytokines in Perioperative Neurocognitive Disorders: A Review in the Context of Anesthetic Care. Biomedicines.

[12] Lu S., Jiang Y., Meng F., Xie X., Wang D., Su Y. (2024). Risk factors for postoperative delirium in patients with Stanford type A aortic dissection: A systematic review and meta-analysis. J. Cardiothorac. Surg..

[13] Wang S., Wang T., Zhao C., Lin D. (2023). Systematic review and meta-analysis of the risk factors for postoperative delirium in patients with acute type A aortic dissection. J. Thorac. Dis..

[14] Staicu R.-E., Vernic C., Ciurescu S., Lascu A., Aburel O.-M., Deutsch P., Rosca E.C. (2025). Postoperative Delirium and Cognitive Dysfunction After Cardiac Surgery: The Role of Inflammation and Clinical Risk Factors. Diagnostics.

[15] Cai S., Li J., Gao J., Pan W., Zhang Y. (2022). Prediction models for postoperative delirium after cardiac surgery: Systematic review and critical appraisal. Int. J. Nurs. Stud..

[16] Wang Y., Zhao X., He X., Shen J., Song J., Lan M., Chen Y.E. (2026). Prediction Models for Postoperative Delirium After Cardiovascular Surgery: A Systematic Review and Critical Appraisal. Nurs. Crit. Care.

[17] Mosharaf M.P., Alam K., Gow J., Mahumud R.A. (2025). Cytokines and inflammatory biomarkers and their association with post-operative delirium: A meta-analysis and systematic review. Sci. Rep..

[18] Lee H., Kim K., Lee C. (2025). Remimazolam-Based Anesthesia and Systemic Inflammatory Biomarkers in Relation to Postoperative Delirium in Elderly Patients: A Retrospective Cohort Study. Medicina.

[19] Mei H., Liao G., Ye B., Wen M., Li J. (2025). Establishment and validation of a nomogram of postoperative delirium in patients undergoing cardiac surgery: A retrospective study of MIMIC-IV. BMC Cardiovasc. Disord..

[20] Song H., Shen L., Sun W., Zhang L., Xue L., Shen Q., Chen Z., Lu X. (2026). Relationship between PCT, CRP, and IL-6 and postoperative delirium in ICU patients and its influencing factors. Blood Cells Mol. Dis..

[21] Brocca A., Virzi G.M., de Cal M., Giavarina D., Carta M., Ronco C. (2017). Elevated Levels of Procalcitonin and Interleukin-6 are Linked with Postoperative Complications in Cardiac Surgery. Scand. J. Surg..

[22] Hansen N., Knopp C.M., Esselmann H., Celano C.M., Derad C., Asendorf T., Chebbok M., Heinemann S., Malik I., Morgado B. (2026). Prediction of postoperative delirium after cardiac surgery by the interplay between preoperative plasma p-tau181 and IL-6 and heart-brain axis related factors: Results from the prospective observational study FINDERI. Mol. Psychiatry.

[23] Wang P., Velagapudi R., Kong C., Rodriguiz R.M., Wetsel W.C., Yang T., Berger M., Gelbard H.A., Colton C.A., Terrando N. (2020). Neurovascular and immune mechanisms that regulate postoperative delirium superimposed on dementia. Alzheimer’s Dement..

[24] Mangalesh S., Dudani S. (2022). Systemic inflammatory response index over neutrophil-lymphocyte ratio and monocyte-lymphocyte ratio: Comparison of prognostic performance in predicting major adverse cardiac events. Ann. Med..

[25] Islam M.M., Satici M.O., Eroglu S.E. (2024). UnrSaveling the clinical significance and prognostic value of the neutrophil-to-lymphocyte ratio, platelet-to-lymphocyte ratio, systemic immune-inflammation index, systemic inflammation response index, and delta neutrophil index: An extensive literature review. Turk. J. Emerg. Med..

[26] Urbanowicz T., Michalak M., Olasińska-Wiśniewska A., Rodzki M., Witkowska A., Gąsecka A., Buczkowski P., Perek B., Jemielity M. (2022). Neutrophil Counts, Neutrophil-to-Lymphocyte Ratio, and Systemic Inflammatory Response Index (SIRI) Predict Mortality after Off-Pump Coronary Artery Bypass Surgery. Cells.

[27] Deng Y., Hua J., Pan H., Zhang J. (2025). Methylene blue reduces postoperative delirium in mice through neuroinflammation suppression and blood-brain barrier repair. Sci. Rep..

[28] Yang T., Velagapudi R., Kong C., Ko U., Kumar V., Brown P., Franklin N.O., Zhang X., Caceres A.I., Min H. (2023). Protective effects of omega-3 fatty acids in a blood-brain barrier-on-chip model and on postoperative delirium-like behaviour in mice. Br. J. Anaesth..

[29] Liu C., Zhang L., Tang W., Zhao S., Li M., Li J., Shao Y. (2024). A nomogram for predicting the risk of postoperative delirium in individuals undergoing cardiovascular surgery. Eur. J. Neurol..

[30] Huang W., Wu Q., Zhang Y., Tian C., Huang H., Wang H., Mao J. (2022). Development and validation of a nomogram to predict postoperative delirium in type B aortic dissection patients underwent thoracic endovascular aortic repair. Front. Surg..

