# Supplementary material for: Clinical Risk Factors and Inflammatory Markers as Predictors of Postoperative Delirium in Patients Undergoing Acute Type A Aortic Dissection Surgery
Source: J Clin Med. 2026 Oct 18;15(20):8090. doi: 10.3390/jcm15208090 (PMC13705684; doi:10.3390/jcm15208090)
Supplement: Supplementary file 1 [file jcm-15-08090-s001.zip › jcm-4561426-supplementary.pdf]

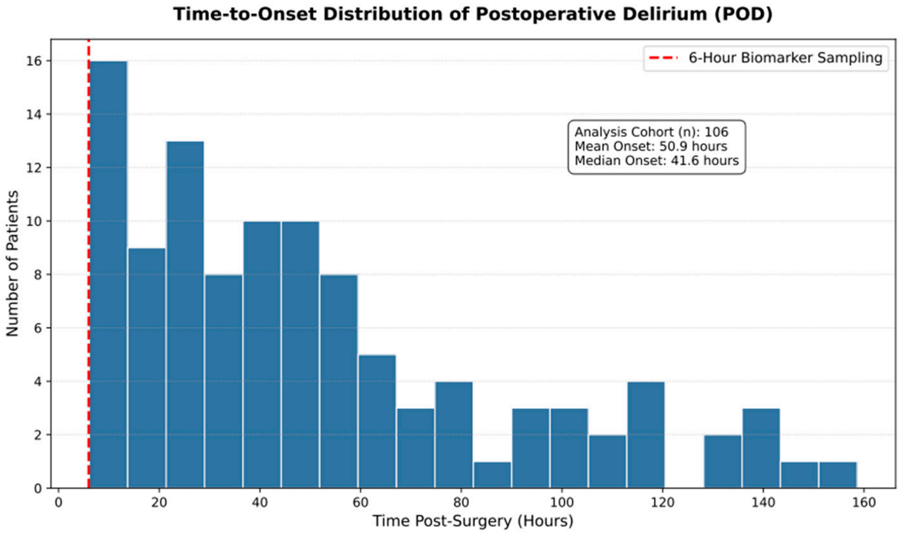

Figure S1. Time-to-Onset Distribution of POD. The histogram illustrates the temporal distribution of initial POD onset among all patients who developed delirium (n = 106). The vertical dashed line indicates the 6-h postoperative time point at which all predictive biomarker samples were collected (mean onset: 50.9 h; median onset: 41.6 h).

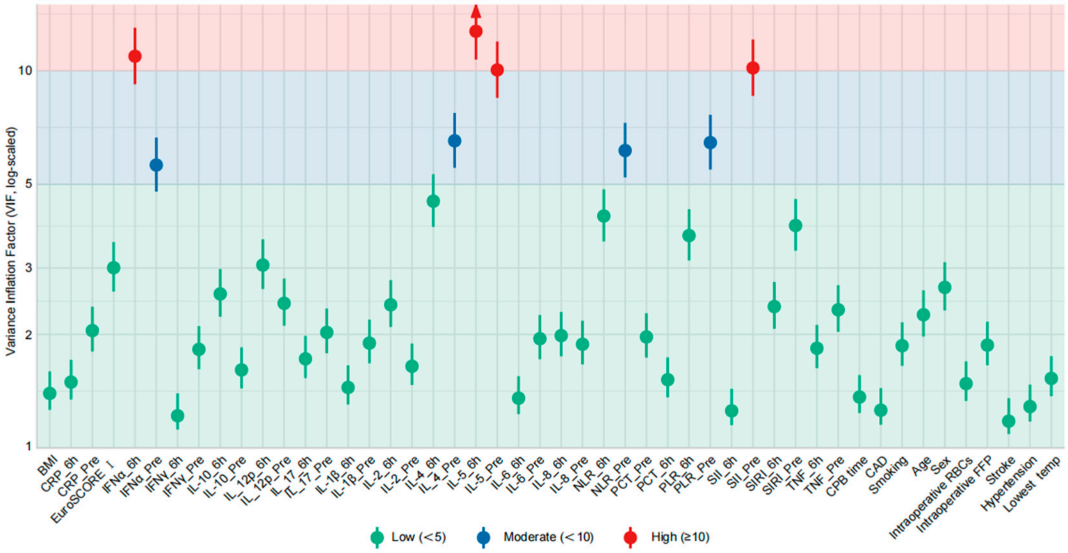

Figure S2. VIF analysis of predictors in ATAAD-POD model (log_{10} scale). Green diamonds = VIF < 5 (acceptable); red zone = VIF ≥ 5 (high collinearity). All VIF < 5 variables retained. See Table 1 for variable definitions.

Table S1. Perioperative Inflammatory Markers and Hematological Indices. Continuous variables are presented as median (interquartile range [IQR]).P-values were calculated using the following tests (superscript numbers indicate the test applied to each variable): [1]Welch Two Sample *t*-test; [2]Wilcoxon rank sum test; [3]Pearson's Chi-squared test; [4]Fisher's exact test.Abbreviations: CRP, C-reactive protein; PCT, procalcitonin; IL, interleukin; IFN, interferon; TNF, tumor necrosis factor; NLR, neutrophil-to-lymphocyte ratio; PLR, platelet-to-lymphocyte ratio; SII, systemic immune-inflammation index; SIRI, systemic inflammation response index; Pre-op, preoperative; Post-op, postoperative; IQR, interquartile range.

Characteristic	Overall (n = 355)	No delirium (n = 249)	Delirium (n = 106)	*P*-value
**Inflammatory Cytokines**				
**CRP, mg/L**				
**Pre-op**	5 (5, 39)	5 (5, 39)	5 (5, 36)	0.939[2]
**Post-op 6h**	116 (90, 150)	116 (90, 150)	115 (90, 149)	0.812[2]
**Post-op d3**	83 (59, 118)	79 (58, 109)	98 (61, 136)	0.007[2]
**Post-op d5**	52 (32, 82)	49 (33, 81)	55 (31, 95)	0.416[2]
**PCT, ng/mL**				
**Pre-op**	0.13 (0.06, 0.49)	0.10 (0.06, 0.38)	0.20 (0.06, 0.72)	0.021[2]
**Post-op 6h**	8 (4, 16)	6 (3, 12)	15 (9, 25)	<0.001[2]
**Post-op d3**	4 (2, 9)	3 (2, 6)	9 (4, 17)	<0.001[2]
**Post-op d5**	1.7 (0.8, 3.7)	1.3 (0.7, 2.8)	3.1 (1.5, 8.2)	<0.001[2]
**IL-1β, pg/mL**				
**Pre-op**	7 (4, 11)	7 (4, 10)	9 (5, 12)	0.072[2]
**Post-op 6h**	5 (2, 9)	5 (2, 9)	4 (2, 8)	0.721[2]
**Post-op d3**	5.0 (1.8, 8.4)	4.0 (1.8, 8.1)	6.3 (2.0, 9.9)	0.058[2]
**Post-op d5**	6 (2, 10)	6 (2, 10)	7 (2, 10)	0.980[2]
**IL-2, pg/mL**				
**Pre-op**	1.69 (1.23, 2.23)	1.64 (1.23, 2.16)	1.77 (1.23, 2.50)	0.144[2]
**Post-op 6h**	1.51 (1.10, 1.94)	1.53 (1.15, 1.98)	1.46 (1.08, 1.87)	0.617[2]
**Post-op d3**	1.38 (1.07, 1.80)	1.41 (1.08, 1.80)	1.27 (1.07, 1.78)	0.187[2]
**Post-op d5**	1.53 (1.11, 2.01)	1.55 (1.20, 2.01)	1.48 (1.07, 2.00)	0.635[2]
**IL-4, pg/mL**				
**Pre-op**	1.09 (0.79, 1.54)	1.09 (0.79, 1.54)	1.11 (0.84, 1.54)	0.686[2]
**Post-op 6h**	0.90 (0.77, 1.53)	0.90 (0.79, 1.53)	0.93 (0.74, 1.53)	0.993[2]
**Post-op d3**	0.93 (0.78, 1.45)	0.91 (0.79, 1.38)	0.95 (0.76, 1.52)	0.678[2]
**Post-op d5**	0.96 (0.79, 1.54)	0.95 (0.79, 1.54)	0.99 (0.79, 1.54)	0.925[2]
**IL-5, pg/mL**				
**Pre-op**	2.22 (1.65, 2.92)	2.16 (1.56, 2.86)	2.29 (1.71, 3.03)	0.185[2]
**Post-op 6h**	1.67 (1.21, 2.38)	1.68 (1.24, 2.59)	1.65 (1.14, 2.13)	0.216[2]
**Post-op d3**	1.70 (1.21, 2.32)	1.66 (1.10, 2.48)	1.71 (1.35, 2.16)	0.489[2]
**Post-op d5**	1.84 (1.30, 2.83)	1.85 (1.30, 2.93)	1.83 (1.28, 2.63)	0.814[2]
**IL-6, pg/mL**				
**Pre-op**	61 (20, 118)	49 (17, 107)	92 (29, 150)	<0.001[2]
**Post-op 6h**	55 (30, 108)	51 (25, 94)	71 (35, 147)	0.001[2]
**Post-op d3**	14 (7, 29)	13 (7, 23)	18 (9, 51)	0.001[2]
**Post-op d5**	14 (8, 28)	13 (7, 25)	18 (8, 37)	0.035[2]
**IL-8, pg/mL**				
**Pre-op**	15 (7, 24)	13 (7, 21)	21 (7, 31)	0.002[2]
**Post-op 6h**	12 (5, 24)	10 (3, 22)	16 (7, 33)	<0.001[2]
**Post-op d3**	7 (2, 17)	7 (2, 13)	12 (5, 30)	<0.001[2]

Characteristic	Overall (n = 355)	No delirium (n = 249)	Delirium (n = 106)	*P*-value
**Post-op d5**	7 (2, 18)	7 (2, 15)	10 (5, 29)	0.001[2]
		**IL-10, pg/mL**		
**Pre-op**	8 (2, 41)	7 (2, 38)	11 (2, 61)	0.066[2]
**Post-op 6h**	6 (3, 14)	5 (3, 11)	9 (4, 32)	<0.001[2]
**Post-op d3**	3 (2, 5)	2 (1, 4)	4 (2, 9)	<0.001[2]
**Post-op d5**	2.4 (1.5, 4.3)	2.2 (1.4, 3.4)	4.0 (1.8, 7.0)	<0.001[2]
		**IL-12p, pg/mL**		
**Pre-op**	1.68 (1.36, 2.00)	1.68 (1.35, 2.01)	1.67 (1.37, 1.95)	0.806[2]
**Post-op 6h**	1.46 (1.24, 1.91)	1.51 (1.24, 2.04)	1.34 (1.25, 1.71)	0.173[2]
**Post-op d3**	1.46 (1.25, 1.91)	1.50 (1.25, 1.95)	1.39 (1.26, 1.88)	0.502[2]
**Post-op d5**	1.61 (1.26, 2.05)	1.68 (1.26, 2.05)	1.41 (1.26, 2.05)	0.393[2]
		**IL-17, pg/mL**		
**Pre-op**	3.8 (2.1, 5.8)	3.4 (2.1, 6.2)	4.1 (2.1, 5.6)	0.694[2]
**Post-op 6h**	2.4 (1.6, 4.8)	2.7 (1.6, 5.5)	2.1 (1.3, 3.7)	0.070[2]
**Post-op d3**	2.5 (1.4, 3.6)	2.5 (1.6, 3.5)	2.1 (1.1, 3.9)	0.320[2]
**Post-op d5**	3.0 (1.6, 4.8)	3.2 (1.6, 4.7)	2.6 (1.5, 6.0)	0.809[2]
		**IFNα, pg/mL**		
**Pre-op**	1.56 (1.20, 2.02)	1.55 (1.18, 1.98)	1.61 (1.31, 2.11)	0.146[2]
**Post-op 6h**	1.31 (1.11, 1.73)	1.31 (1.11, 1.89)	1.31 (1.04, 1.59)	0.249[2]
**Post-op d3**	1.31 (1.11, 1.89)	1.31 (1.11, 1.92)	1.36 (1.11, 1.67)	0.980[2]
**Post-op d5**	1.36 (1.15, 1.97)	1.36 (1.15, 1.97)	1.36 (1.15, 1.88)	0.768[2]
		**IFNγ, pg/mL**		
**Pre-op**	4.7 (2.7, 6.6)	4.5 (2.9, 6.5)	4.8 (2.5, 6.7)	0.919[2]
**Post-op 6h**	2.9 (2.1, 5.6)	3.1 (2.1, 5.7)	2.5 (1.9, 5.4)	0.157[2]
**Post-op d3**	3.3 (2.1, 6.0)	3.4 (2.1, 6.0)	2.8 (2.1, 5.7)	0.508[2]
**Post-op d5**	3.6 (2.1, 6.8)	3.7 (2.1, 7.0)	3.3 (2.1, 6.2)	0.237[2]
		**TNF, pg/mL**		
**Pre-op**	2.20 (1.69, 3.31)	2.20 (1.67, 3.19)	2.31 (1.86, 3.33)	0.215[2]
**Post-op 6h**	1.86 (1.43, 2.20)	1.91 (1.36, 2.20)	1.86 (1.43, 2.20)	0.873[2]
**Post-op d3**	1.76 (1.33, 2.20)	1.83 (1.33, 2.20)	1.72 (1.33, 2.20)	0.863[2]
**Post-op d5**	1.9 (1.4, 2.5)	1.9 (1.4, 2.5)	1.9 (1.4, 2.4)	0.312[2]
		**Hematological Indices**		
		**NLR**		
**Pre-op**	12 (8, 18)	12 (7, 18)	13 (9, 19)	0.243[2]
**Post-op 6h**	25 (19, 33)	25 (20, 35)	23 (17, 31)	0.030[2]
**Post-op d3**	23 (16, 32)	22 (14, 31)	25 (19, 33)	0.010[2]
**Post-op d5**	9 (6, 14)	8 (6, 11)	12 (7, 18)	<0.001[2]
		**PLR**		
**Pre-op**	184 (119, 259)	187 (120, 258)	166 (118, 265)	0.690[2]
**Post-op 6h**	146 (91, 226)	162 (104, 238)	114 (64, 203)	<0.001[2]
**Post-op d3**	165 (109, 236)	169 (116, 238)	151 (95, 233)	0.167[2]
**Post-op d5**	95 (68, 133)	93 (69, 131)	95 (65, 143)	0.793[2]
		**SII**		
**Pre-op**	1835 (1091, 3030)	1874 (1135, 3065)	1676 (1044, 2897)	0.483[2]
**Post-op 6h**	1643 (925, 2539)	1916 (1079, 2846)	1144 (614, 2125)	<0.001[2]
**Post-op d3**	1595 (1090, 2396)	1658 (1128, 2474)	1520 (954, 2355)	0.089[2]
**Post-op d5**	849 (600, 1328)	849 (644, 1272)	850 (567, 1458)	0.912[2]
		**SIRI**		
**Pre-op**	6.9 (4.3, 11.2)	6.6 (4.4, 11.8)	7.5 (4.3, 10.3)	0.947[2]
**Post-op 6h**	14 (9, 23)	16 (9, 25)	12 (8, 17)	0.001[2]

Characteristic	Overall (n = 355)	No delirium (n = 249)	Delirium (n = 106)	*P*-value
**Post-op d3**	8 (6, 14)	8 (6, 14)	8 (6, 13)	0.754[2]
**Post-op d5**	5.9 (3.8, 9.8)	5.5 (3.7, 8.8)	8.2 (4.1, 12.2)	0.012[2]

Table S2. Multivariable Logistic Regression Analysis for POD

Characteristic	Coefficient (β)	SE	β 95% CI	Adjusted OR	95% CI	P-value
Intercept	-7.0945	1.826	—	—	—	<0.001
Age	0.033	0.018	-0.003– 0.068	1.03	1.00–1.07	0.073
SIRI_6h	-0.628	0.248	-1.115– 0.141	0.53	0.33–0.87	0.011
PLR_6h	-0.355	0.223	-0.792– 0.082	0.70	0.45–1.09	0.111
IL10_6h	-0.035	0.147	-0.323– 0.252	0.97	0.72–1.29	0.809
IL8_6h	-0.218	0.159	-0.529– 0.094	0.80	0.59–1.10	0.171
IL8_pre	0.054	0.166	-0.272– 0.380	1.06	0.76–1.46	0.745
IL6_6h	0.527	0.160	0.213– 0.840	1.69	1.24–2.32	<0.001
IL6_pre	0.208	0.140	-0.066– 0.482	1.23	0.94–1.62	0.136
PCT_6h	1.296	0.214	0.877– 1.714	3.65	2.40–5.55	<0.001
EuroSCORE I	0.167	0.132	-0.09– 0.424	1.18	0.91–1.53	0.205
CPB time	0.004	0.004	-0.003– 0.011	1.00	1.00–1.01	0.274

Note: Total cohort n = 355, Event n = 106. Abbreviations: OR = Odds Ratio, CI = Confidence Interval.

Table S3 Clinical Utility Across Decision Thresholds

Threshold	Sensitivity	Specificity	PPV	NPV	High-Risk Patients n (%)
≥ 15%	93.4%	56.2%	47.6%	95.2%	208/355 (58.6%)
≥ 20%	91.5%	63.5%	51.6%	94.6%	188/355 (53.0%)
≥ 25%	88.7%	69.5%	55.3%	93.5%	170/355 (47.9%)

Table S4. Comprehensive Comparison of Baseline Characteristics, Intraoperative Factors, and Postoperative Outcomes Between Included Patients and Patients Excluded Due to Missing Data.

Characteristic	Included Patients (n = 355)	Excluded Patients (n = 108)	*P*-value
**Demographics**			
**Age, years**	55 ± 11	56 ± 12	0.526[1]
**Sex (male), n (%)**	266 (74.9%)	81 (75.0%)	0.988[2]
**BMI, kg/m^2**	24.8 (22.9, 27.1)	24.6 (22.5, 27.1)	0.277[3]
**Comorbidities**			
**Smoking, n (%)**	173 (48.7%)	54 (50.0%)	0.818[2]
**Hypertension, n (%)**	232 (65.4%)	68 (63.0%)	0.649[2]
**T2DM, n (%)**	9 (2.5%)	3 (2.8%)	>0.999[4]
**CAD, n (%)**	11 (3.1%)	9 (8.3%)	0.028[4]
**Stroke, n (%)**	15 (4.2%)	5 (4.6%)	0.792[4]

**Symptom-to-surgery interval, hours**	20 (11, 36)	22 (11, 53)	0.138[3]
**EuroSCORE Ⅰ**	8.00 (8.00, 10.00)	9.00 (8.00, 10.00)	0.167[3]
**Extent of anatomical involvement**			
**Brachiocephalic trunk, n (%)**	240 (67.6%)	54 (50.0%)	<0.001[2]
**Left subclavian artery, n (%)**	255 (71.8%)	55 (50.9%)	<0.001[2]
**Left common carotid artery, n (%)**	230 (64.8%)	54 (50.0%)	0.006[2]
**Celiac trunk, n (%)**	86 (24.2%)	18 (16.7%)	0.099[2]
**Superior mesenteric artery, n (%)**	55 (15.5%)	13 (12.0%)	0.374[2]
**Inferior mesenteric artery, n (%)**	39 (11.0%)	10 (9.3%)	0.610[2]
**Unilateral renal artery, n (%)**	140 (39.4%)	42 (38.9%)	0.919[2]
**Bilateral renal artery, n (%)**	21 (5.9%)	8 (7.4%)	0.575[2]
**Coronary artery, n (%)**	31 (8.7%)	16 (14.8%)	0.067[2]
**Operative Factors**			
CPB time, min	152 (135, 179)	159 (136, 188)	0.143[3]
ACC time, min	89 (75, 106)	92 (75, 111)	0.424[3]
Lowest nasopharyngeal temp, °C	27.70 (26.50, 28.20)	28.00 (26.70, 28.25)	0.150[3]
Intraoperative RBCs, U	4.00 (2.00, 6.00)	4.00 (2.00, 6.00)	0.642[3]
Intraoperative FFP, mL	400 (400, 600)	600 (400, 600)	0.134[3]
**Postoperative Outcomes**			
Delirium, n (%)	106 (29.9%)	27 (25.0%)	0.328[2]
Mechanical ventilation, h	26 (13, 57)	19 (12, 50)	0.071[3]
ICU LOS, d	5 (4, 8)	4 (2, 6)	<0.001[3]
Hospital LOS, d	17 (14, 21)	17 (13, 21)	0.266[3]
In-hospital mortality, n (%)	23 (6.5%)	9 (8.3%)	0.506[2]

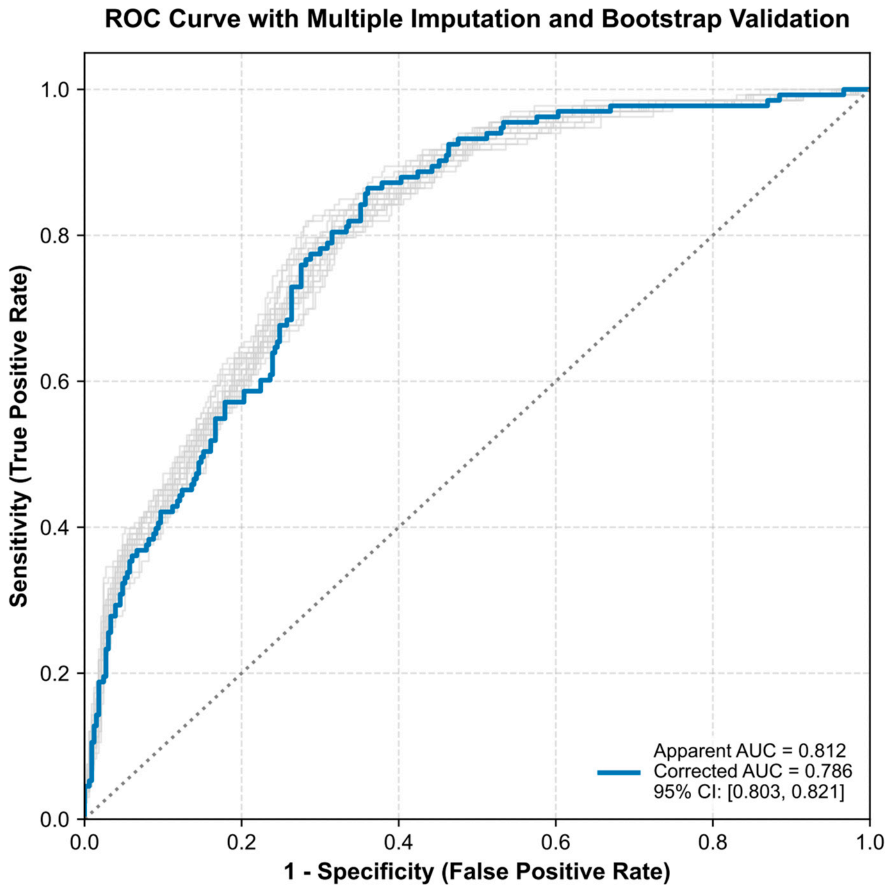

Figure S3. ROC curve with bootstrap internal validation for the multiple-imputation sensitivity analysis model. The apparent AUC is 0.812, and the optimism-corrected AUC yielded by the multiple-imputation sensitivity analysis is 0.786.

Table S5. Association Between SIRI_6h and POD Across Various Model Specifications

Model Specification	Odds Ratio (OR)	95% Confidence Interval (CI)	p-value
Unadjusted Model (Bivariable)	0.547	0.395–0.757	<0.0001
Fully Adjusted Multivariable Model	0.645	0.429–0.968	0.0344
Sensitivity Analysis 1 (Excluding PLR_D1)	0.571	0.391–0.834	0.0038
Sensitivity Analysis 2 (Hematological & Clinical Only)	0.617	0.417–0.912	0.0154

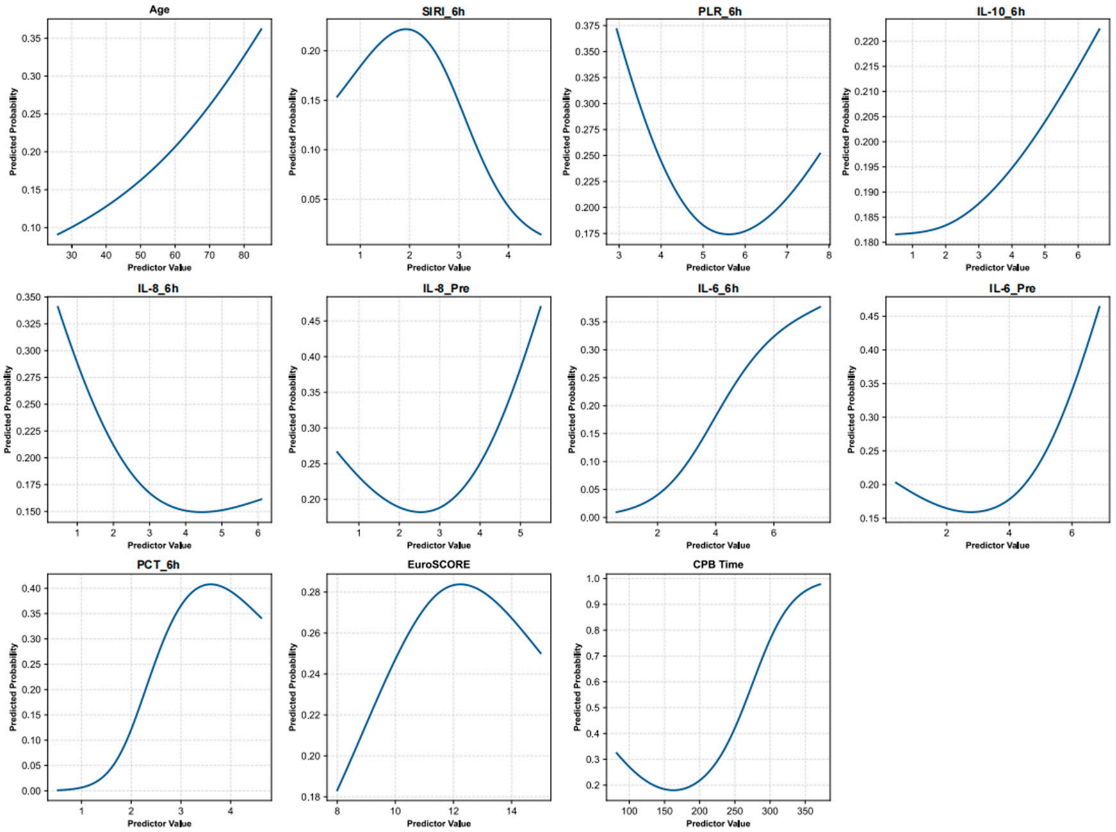

Figure S4. RCS curves capturing the complex non-linear trajectories between continuous predictors and predicted probability. Exploratory RCS modeling (Figure S4) illustrates the adjusted associations and non-linear shapes for individual predictors, including Age, SIRI_6h, PLR_6h, IL-10_6h, IL-8_6h, IL-8_Pre, IL-6_6h, IL-6_Pre, PCT_6h, EuroSCORE, and CPB Time. The y-axis represents the predicted probability, and the x-axis represents the respective predictor values.

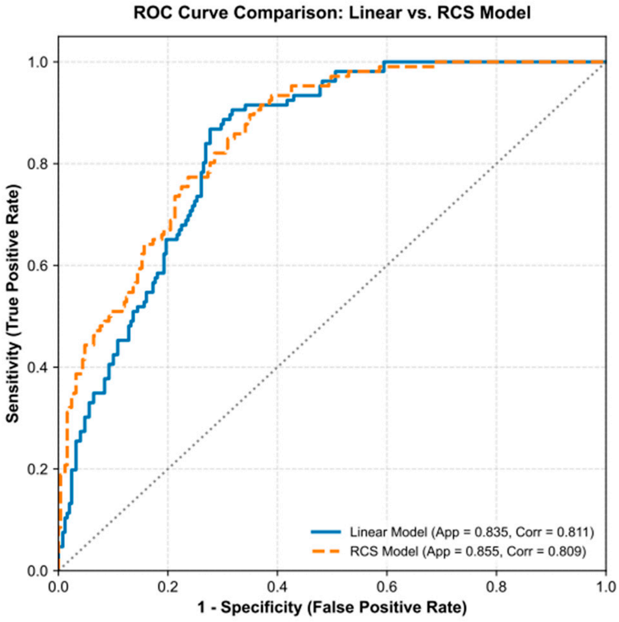

Figure S5. ROC curve comparison between the linear model and the RCS model. The blue solid line represents the linear model (apparent AUC = 0.835,

bias-corrected AUC = 0.811 following a 1000-resample internal bootstrap validation), while the orange dashed line represents the full non-linear RCS model (apparent AUC = 0.855, bias-corrected AUC = 0.809).

Table S6. A full table of selection proportions for all 40 candidate variables

Variable	Selection_Proportion
IL10_6h	1
PLR_6h	1
PCT_6h	1
IL6_6h	0.997
Age	0.994
IL8_6h	0.994
SIRI_6h	0.99
IL8_pre	0.988
IL6_pre	0.966
NLR_6h	0.954
CPB	0.954
BMI	0.928
EuroscoreI	0.91
CRP_6h	0.896
IL1β_pre	0.884
SIRI_pre	0.845
SII_6h	0.755
IL17_6h	0.696
PCT_pre	0.687
IL17_pre	0.639
IL2_6h	0.606
IL10_pre	0.595
IL12p_6h	0.461
IL2_pre	0.414
IL1β_6h	0.322
TNF_pre	0.287
IL4_6h	0.279
IFNγ_pre	0.251
IFNγ_6h	0.244
IL12p_pre	0.18
TNF_6h	0.115
Lowest nasopharyngeal temp	0.096
Intraoperative RBCs	0.093
CRP_pre	0.022
Intraoperative FFP	0.015
Smoking	0.002
Stroke	0
CAD	0
Hypertension	0

Sex (male)	0

TRIPOD Checklist

Item	Section/Topic	Checklist Item Description	Location in Manuscript	Reporting Status/Notes
1	Title	Identify the study as developing and/or validating a multivariable prediction model, the target population, and the outcome to be predicted.	Page 1, Title	As required: '...to develop a predictive nomogram for early POD risk stratification' in patients undergoing ATAAD repair.
2	Abstract	Provide a summary of objectives, study design, setting, participants, sample size, predictors, outcome, statistical analysis, results, and conclusions.	Page 1, Abstract	Structured abstract covers all required elements: objectives, methods, results (AUC 0.835), conclusions.
3a	Introduction: Background	Explain the medical context (disease, patient population, decision about prognosis or diagnosis) and rationale for developing or validating the prediction model.	Page 3, Section 1	Clinical context of POD after ATAAD repair and the inflammatory hypothesis are described.
3b	Introduction: Objectives	Specify the study objectives, including whether the study describes the development or validation of a prediction model.	Page 3–4, Section 1 (last paragraph)	Objective explicitly stated: '...develop and internally validate an exploratory clinical prediction model for POD...'
4a	Methods: Source of data	Describe the study design and specify the study centres and locations, including whether this is a retrospective or prospective collection of data.	Page 4, Section 2.1	Retrospective, single-centre observational study; First Affiliated Hospital of Xi'an Jiaotong University.
4b	Methods: Data collection	Describe methods for data collection and follow-up, and indicate whether data were collected specifically for the study or from an existing database.	Page 4–5, Sections 2.1, 2.4	Data extracted from electronic medical records; consecutive patients, March 2020–December 2022.
5a	Methods: Participants	Specify key elements of the study setting (e.g., primary care, secondary	Page 4, Section 2.1	Single-centre; inclusion: consecutive ATAAD patients undergoing

		care, general population) and participants (e.g., referral pattern, inclusion and exclusion criteria).		emergent total arch replacement; exclusion criteria listed.
5b	Methods: Participants	Give the rationale for considering potential participants for the study.	Page 4, Section 2.1	Patients undergoing ATAAD repair were considered for POD risk modelling.
5c	Methods: Participants	Give the rationale for the choice of the control group, if applicable.	N/A	Prognosis (not diagnostic) study; no separate control group.
6a	Methods: Outcome	Clearly define the outcome that is being predicted, including the diagnostic or prognostic classification or threshold, and the time interval.	Page 4, Section 2.3	POD defined by CAM-ICU; assessed twice daily from postoperative 6h to day 7.
6b	Methods: Outcome	Detail the methods and timing of assessment of the outcome and (if applicable) the definition and management of any treatments administered after baseline.	Page 4-5, Section 2.3	CAM-ICU performed by trained ICU physicians, blinded to biomarker results; RASS assessed first.
7a	Methods: Predictors	Clearly define all predictors used in the considered models, including how and when they were measured, and indicate whether they were assessed blinded to other predictors and the outcome.	Page 5–6, Section 2.4	48 candidate variables; inflammatory markers at pre-op, 6h, POD3, POD5; lab personnel blinded; log-transformation applied.
7b	Methods: Predictors	Describe and justify any assumptions made about future predictor values, if applicable.	Page 6, Section 2.5.1	Preoperative and 6h postoperative markers assumed available at prediction timepoint.
8	Methods: Sample size	Explain how the study size was derived, including whether and how the sample size was determined in relation to the number of events (for development studies) and anticipated performance of the model (for external validation).	Page 4, Section 2.1	No a priori calculation; sample size determined by maximum consecutive eligible cohort. EPV = 106/11 = 9.6. Limitation acknowledged in Section 4.

9	Methods: Missing data	Describe how missing data were handled. If necessary, give reasons for omitting participants from the analysis and explain the impact of missing data on the representativeness of the reported sample.	Page 4–5, Sections 2.1, 2.6	Complete-case analysis; 108 of 181 excluded for missing data. Sensitivity analysis with multiple imputation (AUC 0.797).
10a	Methods: Analytic approach	Describe how predictors were handled in the analyses, including any transformation, coding, or standardisation.	Page 6, Section 2.5.1	Log2-transformation for skewed inflammatory markers; clinical variables retained as continuous or categorical.
10b	Methods: Analytic approach	Specify the type of model and the procedure for selecting the predictors for inclusion in the final model.	Page 6, Section 2.5.1–2.5.2	Multivariable logistic regression; two-stage selection using VIF (<5) and Boruta algorithm.
10c	Methods: Analytic approach	Describe any attempts to examine the correlation between candidate predictors and, if relevant, any strategies used to address this.	Page 6, Section 2.5.2	VIF <5 applied to address multicollinearity among candidate predictors.
10d	Methods: Analytic approach	Give the rationale and detailed description of the approach used for (internal) validation.	Page 6, Section 2.5.3	Internal validation via 1000 bootstrap resamples; optimism correction applied (corrected C-index 0.811).
10e	Methods: Analytic approach	Describe all statistical methods for model examination and presentation.	Page 6, Section 2.5.3–2.5.4	AUC, calibration plots (calibration slope 1.0000), Hosmer–Lemeshow test, DCA, sensitivity/specificity analysis.
11	Methods: Risk groups	Give the rationale for and detailed description of any risk groups or strata examined, if applicable.	N/A	No risk strata defined; continuous risk prediction via nomogram.
12	Methods: Development vs validation	For validation studies, give the rationale and detailed description of any differences between the data source for development and validation, if applicable.	Page 4, Section 2.1	This study includes only development and internal validation; no external validation dataset.

13a	Results: Participants	Report the numbers of individuals at each stage of the study (e.g., participants recruited, eligible, included in analysis).	Page 5, Figure 1	536 screened; 355 included in final analysis (flow chart provided).
13b	Results: Participants	Report reasons for ineligibility and non-participation.	Page 5, Figure 1	Exclusion details: missing assessments (n = 44), pre-existing cognitive disorders (n = 2), intraoperative death (n = 11), ICU <24h (n = 16), missing data (n = 108).
14a	Results: Descriptive statistics	Report the characteristics of the participants, including demographic, clinical, and predictor data.	Page 7, Table 1	Baseline characteristics presented by POD status (POD vs. non-POD).
14b	Results: Descriptive statistics	For validation studies, report the unadjusted association between the outcome and each predictor.	N/A (development study)	Unadjusted associations reported in Table 1.
15a	Results: Model specification	Give the full prediction model, including the nature and transformation of all predictors, with enough detail so that the model can be implemented by others.	Page 8, Section 3.4; Supplementary Table S2	Full regression equation with 11 coefficients and intercept provided; web calculator available.
15b	Results: Model specification	Report model performance measures (with CIs) for the development dataset and, if applicable, for any validation datasets.	Page 8, Section 3.5; Figure 4B–D	AUC 0.835 (95% CI 0.794–0.876); optimism-corrected C-index 0.811.
16	Results: Model performance	Report model performance measures, including discrimination and calibration, with CIs.	Page 8, Section 3.5	Discrimination (AUC), calibration (slope 1.0000, intercept 0.0028), and DCA all reported with CIs.
17	Results: Model updating	If done, report the results of any model updating, including the updated model, and subsequent performance measures, if applicable.	N/A	No model updating performed.

18	Discussion: Limitations	Discuss any limitations of the study (e.g., non-representative sample, high missing data, errors in measurements, small sample size, few events per predictor, inappropriate choices regarding predictors, model selection, validation, and presentation).	Page 10, Section 4	Single-centre retrospective design; EPV = 9.6; complete-case analysis; no external validation—all acknowledged.
19a	Discussion: Interpretation	Give an overall interpretation of the results, considering objectives, limitations, and results from other relevant studies.	Page 9–10, Section 4	Model provides a preliminary foundation for early risk stratification; compared with existing literature.
19b	Discussion: Interpretation	Describe the potential clinical usefulness of the model.	Page 10, Section 4	Web-based calculator developed to facilitate bedside implementation; pending external validation.
20	Other information: Abstract	Provide information about the availability of the full prediction model, including predictor definitions and model equation, or an accessible version of the model (e.g., web calculator).	Page 10, Section 3.4; Supplementary Table S2	Full model equation and web calculator link provided.
21	Other information: Funding	Give the source of funding and the role of the funders for the study.	Page 11, Funding	Funding sources stated.
22	Other information: Conflicts of Interest	Declare any conflicts of interest.	Page 11, Conflicts of Interest	Conflicts of interest declared.

Table S7a. PROBAST Domain-Level Assessment

PROBAST Domain	Development: ROB	Internal validation: ROB	Rationale (key signalling questions addressed)
1. Participants	Low	Low	Consecutive patients with ATAAD undergoing emergent total arch replacement at a single tertiary centre (Mar 2020–Dec 2022). Clear, prespecified inclusion/exclusion criteria; flow chart

			provided (Figure 1); target population matches review PICO.
2. Predictors	Low	Low	All candidate predictors defined a priori from EMR; measured at fixed time points (preoperative and strictly at 6h postoperatively); laboratory personnel blinded to POD status; log-transformation for skewed inflammatory markers; no data-driven predictor modification during bootstrap validation.
3. Outcome	Low	Low	POD assessed by CAM-ICU, a gold-standard critical-care diagnostic tool; trained ICU physicians performed assessments; twice-daily surveillance from postoperative 6h to day 7; nurses blinded to biomarker results; blood sampling preceded POD onset in all cases, ensuring correct temporal ordering.
4. Analysis	High	Low	**Development:** EPV = 106/11 ≈ 9.6 (< 10), with no formal a priori sample-size calculation, leading to High ROB (signalling question 4.2). Mitigating factors: VIF <5 to control collinearity; Boruta wrapper selection; complete-case analysis with multiple-imputation sensitivity analysis (AUC 0.797). **Internal validation:** 1000 bootstrap resamples with optimism correction appropriately reported (apparent AUC 0.835 → optimism-corrected C-index 0.811); calibration slope 1.00; DCA performed.
Overall ROB	High	Low	Overall ROB is High only when all domains are Low; Domain 4 (Analysis) is High at development, so overall development ROB = High. Internal validation ROB = Low.
Applicability	Low concern	Low concern	All four domains raise low concern regarding applicability to the review question (POD risk prediction in adult patients undergoing ATAAD surgical repair).

Table S7b. PROBAST Step-Level Summary

Step	Overall Risk of Bias	Rationale
Development	High	Domains 1–3 Low. **Domain 4 (Analysis) High** because EPV = 9.6 (< 10): 106 POD events and 11 model parameters, with no formal a priori sample-size calculation (Section 2.1, Discussion). VIF control, Boruta selection and optimism correction mitigate but do not eliminate this risk.
Internal validation	Low	Same cohort; 1000-bootstrap resampling with optimism correction appropriately described and reported (apparent AUC 0.835; optimism-corrected C-index 0.811; calibration slope 1.00); no external temporal or geographic validation available.
